# Supplementary material for: Associations between participation in organised physical activity in the school or community outside school hours and neighbourhood play with child physical activity and sedentary time: a cross-sectional analysis of primary school-aged children from the UK
Source: BMJ Open. 2017 Sep 14;7(9):e017588. doi: 10.1136/bmjopen-2017-017588 (PMC5640140; doi:10.1136/bmjopen-2017-017588)
Supplement: Supplementary file 1 [file bmjopen-2017-017588supp001.pdf]

**Table S1 Frequencies of sport/exercise club attendance and play outside and inside the home and mean activity score by gender in the observed data**

| | | Boys N (%) | Girls N (%) | Chi-squared p-value for association |
|---|---|---|---|---|
| **Frequency child attends sport/exercise club at school** | Never | 144 (25.9) | 194 (29.4) | <0.001 |
| | 1-2 days/week | 235 (42.3) | 318 (48.2) | |
| | 3-4 days/week | 90 (16.2) | 106 (16.1) | |
| | 5 days/week | 86 (15.5) | 42 (6.4) | |
| **Frequency child attends sport/exercise club outside of school** | Never | 85 (15.3) | 165 (25.0) | <0.001 |
| | 1-2 days/week | 289 (52.1) | 321 (48.7) | |
| | 3-4 days/week | 121 (21.8) | 132 (20.0) | |
| | 5 days/week | 60 (10.8) | 41 (6.2) | |
| **Frequency child plays with friends/family outside near home** | Never | 36 (6.6) | 40 (6.1) | 0.14 |
| | 1-2 days/week | 187 (34.1) | 219 (33.4) | |
| | 3-4 days/week | 142 (25.9) | 207 (31.6) | |
| | 5 days/week | 184 (33.5) | 190 (29.0) | |
| **Frequency child plays with friends/family in home/garden** | Never | 53 (9.7) | 62 (9.5) | 0.76 |
| | 1-2 days/week | 184 (33.7) | 230 (35.2) | |
| | 3-4 days/week | 142 (26.0) | 179 (27.4) | |
| | 5 days/week | 167 (30.6) | 182 (27.9) | |

| | Boys | | Girls | | T-test p-value for gender difference |
|---|---|---|---|---|---|
| | N | Mean (SD) | N | Mean (SD) | |
| **Activity frequency score** | 542 | 6.15 (2.42) | 651 | 5.65 (2.16) | <0.001 |

**Table S2 Child characteristics by frequencies of sport/exercise club attendance and play outside and inside the home and activity score in the observed data**

| | | Age (years) | | BMI z score | | IMD score | |
|---|---|---|---|---|---|---|---|
| | | Mean (SD) | N | Mean (SD) | N | Mean (SD) | N |
| **Frequency child attends sport/exercise club at school** | Never<br>1-2 days/week<br>3-4 days/week<br>5 days/week | 9.03 (0.38)<br>9.05 (0.43)<br>9.00 (0.45)<br>8.99 (0.39) | 1215 | 0.43 (1.14)<br>0.35 (1.05)<br>0.29 (1.02)<br>0.27 (1.13) | 1202 | 17.30 (15.04)<br>15.54 (14.02)<br>14.15 (11.07)<br>16.66 (15.10) | 1197 |
| P-value for difference between categories* | | 0.31 | | 0.36 | | 0.07 | |
| **Frequency child attends sport/exercise club outside of school** | Never<br>1-2 days/week<br>3-4 days/week<br>5 days/week | 9.01 (0.40)<br>9.01 (0.43)<br>9.05 (0.41)<br>9.12 (0.39) | 1214 | 0.47 (1.07)<br>0.37 (1.09)<br>0.23 (1.07)<br>0.30 (1.06) | 1201 | 20.03 (16.06)<br>16.02 (14.12)<br>12.30 (10.25)<br>14.26 (13.64) | 1196 |
| P-value for difference between categories* | | 0.05 | | 0.09 | | <0.001 | |
| **Frequency child plays with friends/family outside near home** | Never<br>1-2 days/week<br>3-4 days/week<br>5 days/week | 8.96 (0.47)<br>9.01 (0.42)<br>9.05 (0.41)<br>9.05 (0.40) | 1205 | 0.48 (1.24)<br>0.31 (1.06)<br>0.39 (1.07)<br>0.33 (1.08) | 1192 | 17.60 (16.02)<br>15.20 (13.83)<br>14.66 (12.48)<br>17.66 (15.08) | 1187 |
| P-value for difference between categories* | | 0.23 | | 0.58 | | 0.01 | |
| **Frequency child plays with friends/family in home/garden** | Never<br>1-2 days/week<br>3-4 days/week<br>5 days/week | 8.97 (0.38)<br>9.02 (0.45)<br>9.06 (0.41)<br>9.03 (0.39) | 1199 | 0.50 (1.11)<br>0.29 (1.09)<br>0.40 (1.07)<br>0.32 (1.07) | 1186 | 18.54 (15.94)<br>15.85 (13.95)<br>14.24 (12.79)<br>16.85 (14.55) | 1181 |
| P-value for difference between categories* | | 0.31 | | 0.23 | | 0.02 | |
| | | Correlation | P-value | Correlation | P-value | Correlation | P-value |
| **Activity frequency score** | | 0.04 | 0.13 | -0.05 | 0.07 | -0.07 | 0.02 |

**Table S3 Pairwise comparisons of children's activity attendance frequencies in the multiple imputation data (N=1223)[*]**

| | | **Frequency child attends sport/exercise club at school (%)** | | **Frequency child attends sport/exercise club outside of school (%)** | | **Frequency child plays with friends/family outside near home (%)** | | **Frequency child plays with friends/family in home/garden (%)** | |
|---|---|---|---|---|---|---|---|---|---|
| | | Up to 2 days/week | 3 or more days/week | Up to 2 days/week | 3 or more days/week | Up to 2 days/week | 3 or more days/week | Up to 2 days/week | 3 or more days/week |
| **Frequency child attends sport/exercise club at school** | Up to 2 days/week | | | 76.3 | 23.7 | 42.7 | 57.3 | 47.3 | 52.7 |
| | 3 or more days/week | | | 55.8 | 44.2 | 33.2 | 66.8 | 36.0 | 64.0 |
| P for association | | | | <0.001 | | 0.003 | | 0.001 | |
| **Frequency child attends sport/exercise club outside of school** | Up to 2 days/week | 79.0 | 21.0 | | | 40.2 | 59.8 | 46.8 | 53.2 |
| | 3 or more days/week | 59.7 | 40.3 | | | 40.0 | 60.0 | 38.2 | 61.8 |
| P for association | | <0.001 | | | | 0.94 | | 0.006 | |
| **Frequency child plays with friends/family outside near home** | Up to 2 days/week | 78.0 | 22.0 | 71.0 | 29.0 | | | 65.1 | 34.9 |
| | 3 or more days/week | 70.3 | 29.7 | 70.8 | 29.2 | | | 30.3 | 69.7 |
| P for association | | 0.003 | | 0.94 | | | | <0.001 | |
| **Frequency child plays with friends/family in home/garden** | Up to 2 days/week | 78.3 | 21.7 | 74.9 | 25.1 | 59.0 | 41.0 | | |
| | 3 or more days/week | 69.4 | 30.6 | 67.6 | 32.4 | 25.2 | 74.8 | | |
| P for association | | 0.001 | | 0.006 | | <0.001 | | | |
| **Total** | | **73.4** | **26.6** | **70.8** | **29.2** | **40.2** | **59.8** | **44.3** | **55.7** |

[*] Percentages presented are the proportions of children in each row that belong to each of the categories of child activity variables listed along the top of the table.

**Table S4 Mean difference in the children's average sedentary minutes per day associated with different activities for those with complete data (N=987)***

| Exposure | | Sedentary time (minutes/day): mean difference (95% confidence interval) | | | | | | P for gender interaction |
|---|---|---|---|---|---|---|---|---|
| | | All (N=987) | | Boys (N=439) | | Girls (N=548) | | |
| | | Model 1 | Model 2 | Model 1 | Model 2 | Model 1 | Model 2 | |
| Frequency child attends sport/exercise club at school | Never (ref) | 0 | 0 | 0 | 0 | 0 | 0 | 0.45 |
| | 1-2 days/week | -0.29 (-21.3, 20.7) | 0.0 (-21.2, 21.3) | 14.7 (-10.1, 39.5) | 15.9 (-9.2, 41.0) | -11.4 (-39.7, 16.8) | -11.9 (-41.1, 17.3) | |
| | 3-4 days/week | -26.3 (-40.8, -11.8) | -24.1 (-37.8, -10.4) | -13.8 (-31.5, 4.0) | -9.9 (-29.3, 9.4) | -36.0 (-59.5, -12.4) | -35.4 (-59.0, -11.7) | |
| | 5 days/week | -16.0 (-35.3, 3.3) | -16.1 (-34.4, 2.3) | -7.1 (-29.7, 15.4) | -3.1 (-26.9, 20.6) | -22.0 (-54.6, 10.7) | -29.8 (-69.2, 9.66) | |
| P for trend | | 0.01 | 0.009 | 0.19 | 0.40 | 0.02 | 0.02 | |
| Frequency child attends sport/exercise club outside of school | Never (ref) | 0 | 0 | 0 | 0 | 0 | 0 | 0.73 |
| | 1-2 days/week | 16.1 (-7.5, 39.7) | 20.0 (-7.0, 47.0) | 24.6 (-3.4, 52.7) | 31.2 (0.6, 61.8) | 10.8 (-19.3, 41.0) | 12.9 (-20.0, 45.9) | |
| | 3-4 days/week | -6.6 (-23.2, 10.1) | 0.1 (-19.6, 19.8) | -3.2 (-28.8, 22.3) | 5.6 (-17.7, 28.8) | -7.6 (-26.3, 11.1) | -2.0 (-26.7, 22.6) | |
| | 5 days/week | -18.4 (-36.5, -0.2) | -13.8 (-33.8, 6.2) | -13.0 (-36.3, 10.4) | -5.9 (-28.8, 16.9) | -20.8 (-46.0, 4.3) | -18.2 (-46.4, 10.0) | |
| P for trend | | 0.009 | 0.05 | 0.02 | 0.03 | 0.08 | 0.28 | |
| Frequency child plays with friends/family outside near home | Never (ref) | 0 | 0 | 0 | 0 | 0 | 0 | 0.68 |
| | 1-2 days/week | 4.5 (-26.7, 35.7) | 6.0 (-27.5, 39.5) | 16.3 (-13.4, 45.9) | 20.2 (-10.1, 50.4) | -5.7 (-55.7, 44.3) | -7.1 (-59.0, 44.9) | |
| | 3-4 days/week | -2.5 (-30.1, 25.0) | -0.6 (-31.6, 30.3) | 8.3 (-23.1, 39.7) | 13.0 (-19.4, 45.4) | -11.9 (-68.1, 44.3) | -13.2 (-71.3, 44.9) | |
| | 5 days/week | -21.6 (-49.0, 5.7) | -21.9 (-50.4, 6.6) | -9.4 (-35.4, 16.6) | -6.4 (-32.8, 20.0) | -32.3 (-78.9, 14.2) | -36.4 (-86.0, 13.1) | |
| P for trend | | <0.001 | <0.001 | 0.08 | 0.08 | 0.009 | 0.01 | |
| Frequency child plays with friends/family in home/garden | Never (ref) | 0 | 0 | 0 | 0 | 0 | 0 | 0.86 |
| | 1-2 days/week | 18.2 (-12.4, 48.8) | 18.8 (-12.4, 50.0) | 19.2 (-13.8, 52.2) | 19.5 (-13.7, 52.8) | 17.2 (-25.4, 59.9) | 17.6 (-25.4, 60.6) | |
| | 3-4 days/week | -4.5 (-27.3, 18.2) | -1.9 (-26.7, 22.9) | 2.8 (-16.3, 21.9) | 4.6 (-15.7, 25.0) | -10.1 (-47.3, 27.0) | -7.5 (-47.1, 32.1) | |
| | 5 days/week | 3.8 (-24.6, 32.2) | 3.6 (-24.6, 31.8) | 10.8 (-27.1, 48.8) | 11.6 (-27.0, 50.2) | -2.2 (-43.0, 38.6) | -3.5 (-45.3, 38.3) | |
| P for trend | | 0.24 | 0.22 | 0.85 | 0.89 | 0.25 | 0.24 | |
| Activity score (per unit) | | -4.9 ( -7.2, -2.7) | -4.7 (-6.9, -2.4) | -3.5 (-6.6, -0.4) | -2.9 (-6.0, 0.2) | -6.3 (-10.7, -2.0) | -6.6 (-11.2, -1.9) | 0.28 |

* Model 1 is adjusted for age and gender; Model 2 is additionally adjusted for BMI and IMD score

**Table S5 Mean difference in the children's average MVPA minutes per day associated with different activities for those with complete data (N=987)[*]**

| Exposure | | Moderate-to-vigorous physical activity (minutes/day): mean difference (95% confidence interval) | | | | | | |
|---|---|---|---|---|---|---|---|---|
| | | All (N=987) | | Boys (N=439) | | Girls (N=548) | | P for gender interaction |
| | | Model 1 | Model 2 | Model 1 | Model 2 | Model 1 | Model 2 | |
| Frequency child attends sport/exercise club at school | Never (ref) | 0 | 0 | 0 | 0 | 0 | 0 | 0.64 |
| | 1-2 days/week | 3.2 (-0.4, 6.8) | 3.1 (-0.5, 6.6) | 3.3 (-3.3, 9.8) | 2.7 (-3.7, 9.2) | 3.1 (-0.5, 6.8) | 3.1 (-0.6, 6.8) | |
| | 3-4 days/week | 6.7 (1.7, 11.8) | 6.4 (1.4, 11.4) | 8.0 (1.0, 15.1) | 7.3 (0.1, 14.4) | 5.7 (0.6, 10.7) | 5.6 (0.5, 10.7) | |
| | 5 days/week | 5.3 (-1.3, 11.9) | 5.1 (-1.5, 11.8) | 6.6 (-1.4, 14.6) | 5.8 (-2.2, 13.8) | 3.2 (-5.0, 11.5) | 3.1 (-5.5, 11.6) | |
| P for trend | | 0.02 | 0.03 | 0.02 | 0.05 | 0.08 | 0.10 | |
| Frequency child attends sport/exercise club outside of school | Never (ref) | 0 | 0 | 0 | 0 | 0 | 0 | 0.53 |
| | 1-2 days/week | 1.8 (-1.9, 5.5) | 1.5 (-2.4, 5.4) | 4.3 (-3.2, 11.9) | 3.6 (-4.4, 11.7) | 0.4 (-3.5, 4.3) | 0.4 (-3.5, 4.4) | |
| | 3-4 days/week | 7.3 (3.0, 11.5) | 6.7 (2.4, 11.0) | 10.5 (2.8, 18.2) | 9.0 (1.4, 16.6) | 5.3 (0.6, 10.0) | 5.5 (0.9, 10.0) | |
| | 5 days/week | 10.5 (4.6, 16.5) | 10.1 (3.9, 16.2) | 14.3 (6.3, 22.3) | 13.4 (4.8, 22.0) | 7.3 (-0.8, 15.3) | 7.3 (-0.6, 15.2) | |
| P for trend | | <0.001 | <0.001 | <0.001 | <0.001 | 0.01 | 0.008 | |
| Frequency child plays with friends/family outside near home | Never (ref) | 0 | 0 | 0 | 0 | 0 | 0 | 0.32 |
| | 1-2 days/week | 0.7 (-4.1, 5.5) | 0.4 (-4.3, 5.0) | 1.2 (-7.4, 9.7) | 0.5 (-8.1, 9.1) | 0.4 (-6.0, 6.9) | 0.3 (-6.3, 6.9) | |
| | 3-4 days/week | 5.2 (-0.8, 11.2) | 4.9 (-1.0, 10.7) | 6.7 (-3.5, 16.9) | 6.0 (-4.0, 15.9) | 4.0 (-3.6, 11.7) | 3.9 (-3.8, 11.7) | |
| | 5 days/week | 9.9 (4.4, 15.4) | 9.7 (4.2, 15.1) | 13.8 (4.6, 23.0) | 13.3 (4.0, 22.5) | 6.5 (-0.5, 13.5) | 6.4 (-0.7, 13.6) | |
| P for trend | | <0.001 | <0.001 | <0.001 | <0.001 | 0.004 | 0.005 | |
| Frequency child plays with friends/family in home/garden | Never (ref) | 0 | 0 | 0 | 0 | 0 | 0 | 0.40 |
| | 1-2 days/week | 2.5 (-0.9, 6.0) | 2.2 (-1.4, 5.8) | 0.6 (-7.0, 8.2) | 0.4 (-7.4, 8.2) | 4.1 (-0.9, 9.2) | 4.0 (-1.1, 9.1) | |
| | 3-4 days/week | 6.6 (2.4, 10.7) | 6.3 (1.8, 10.8) | 6.7 (-1.8, 15.3) | 6.9 (-1.9, 15.7) | 6.5 (0.4, 12.6) | 6.4 (0.1, 12.7) | |
| | 5 days/week | 8.6 (4.6, 12.5) | 8.4 (4.3, 12.6) | 9.3 (1.6, 16.9) | 9.1 (1.0, 17.2) | 7.9 (2.5, 13.3) | 7.8 (2.4, 13.3) | |
| P for trend | | <0.001 | <0.001 | 0.001 | 0.001 | 0.003 | 0.003 | |
| Activity score (per unit) | | 2.13 (1.44, 2.82) | 2.09 (1.37, 2.81) | 2.58 (1.73, 3.43) | 2.49 (1.55, 3.42) | 1.67 (0.86, 2.47) | 1.66 (0.85, 2.48) | 0.07 |

[*] MVPA Moderate-to-vigorous physical activity; Model 1 is adjusted for age and gender; Model 2 is additionally adjusted for BMI and IMD score

**Table S6 Odds ratios for achieving 60 minutes of MVPA per day associated with different activities for those with complete data (N=987)***

| **Exposure** | | **Meeting government guideline: odds ratio (95% confidence interval)** | | | | | | |
|---|---|---|---|---|---|---|---|---|
| | | **All (N=987)** | | **Boys (N=439)** | | **Girls (N=548)** | | **P for gender interaction** |
| | | **Model 1** | **Model 2** | **Model 1** | **Model 2** | **Model 1** | **Model 2** | |
| Frequency child attends sport/exercise club at school | Never (ref) | 1 | 1 | 1 | 1 | 1 | 1 | 0.25 |
| | 1-2 days/week | 1.27 (0.94, 1.71) | 1.25 (0.93, 1.69) | 0.97 (0.58, 1.63) | 0.93 (0.55, 1.55) | 1.57 (1.09, 2.25) | 1.57 (1.09, 2.26) | |
| | 3-4 days/week | 1.77 (1.05, 2.97) | 1.72 (1.03, 2.87) | 1.97 (0.93, 4.18) | 1.86 (0.88, 3.95) | 1.65 (0.93, 2.91) | 1.66 (0.94, 2.92) | |
| | 5 days/week | 1.63 (0.96, 2.77) | 1.61 (0.95, 2.74) | 1.49 (0.75, 2.96) | 1.40 (0.70, 2.77) | 1.73 (0.78, 3.85) | 1.76 (0.78, 3.96) | |
| P for trend | | 0.03 | 0.03 | 0.06 | 0.10 | 0.07 | 0.07 | |
| Frequency child attends sport/exercise club outside of school | Never (ref) | 1 | 1 | 1 | 1 | 1 | 1 | 0.66 |
| | 1-2 days/week | 1.32 (0.91, 1.91) | 1.29 (0.88, 1.88) | 1.53 (0.89, 2.62) | 1.46 (0.83, 2.57) | 1.19 (0.72, 1.97) | 1.21 (0.74, 1.99) | |
| | 3-4 days/week | 2.06 (1.38, 3.07) | 1.97 (1.32, 2.95) | 2.56 (1.32, 4.98) | 2.28 (1.18, 4.40) | 1.77 (1.05, 2.98) | 1.81 (1.09, 3.03) | |
| | 5 days/week | 2.89 (1.76, 4.73) | 2.80 (1.69, 4.63) | 4.13 (1.97, 8.68) | 3.92 (1.82, 8.44) | 2.09 (1.06, 4.12) | 2.14 (1.10, 4.18) | |
| P for trend | | <0.001 | <0.001 | <0.001 | <0.001 | 0.006 | 0.004 | |
| Frequency child plays with friends/family outside near home | Never (ref) | 1 | 1 | 1 | 1 | 1 | 1 | 0.89 |
| | 1-2 days/week | 1.16 (0.70, 1.92) | 1.13 (0.70, 1.85) | 1.03 (0.46, 2.29) | 0.98 (0.43, 2.22) | 1.40 (0.59, 3.30) | 1.41 (0.60, 3.29) | |
| | 3-4 days/week | 1.94 (1.12, 3.35) | 1.89 (1.12, 3.21) | 1.59 (0.68, 3.75) | 1.53 (0.65, 3.62) | 2.43 (0.92, 6.45) | 2.46 (0.94, 6.42) | |
| | 5 days/week | 2.14 (1.26, 3.65) | 2.12 (1.26, 3.57) | 2.01 (0.88, 4.63) | 1.97 (0.85, 4.54) | 2.45 (0.95, 6.36) | 2.49 (0.97, 6.40) | |
| P for trend | | <0.001 | <0.001 | 0.007 | 0.005 | 0.007 | 0.006 | |
| Frequency child plays with friends/family in home/garden | Never (ref) | 1 | 1 | 1 | 1 | 1 | 1 | 0.30 |
| | 1-2 days/week | 1.33 (0.88, 2.00) | 1.30 (0.86, 1.97) | 0.96 (0.51, 1.78) | 0.94 (0.50, 1.77) | 1.92 (0.90, 4.11) | 1.94 (0.91, 4.12) | |
| | 3-4 days/week | 1.67 (1.10, 2.54) | 1.64 (1.07, 2.52) | 1.33 (0.66, 2.69) | 1.36 (0.66, 2.82) | 2.15 (0.96, 4.79) | 2.16 (0.97, 4.80) | |
| | 5 days/week | 1.86 (1.21, 2.86) | 1.85 (1.20, 2.86) | 1.68 (0.86, 3.25) | 1.67 (0.84, 3.32) | 2.18 (1.04, 4.60) | 2.20 (1.04, 4.62) | |
| P for trend | | 0.002 | 0.002 | 0.008 | 0.008 | 0.06 | 0.06 | |
| Activity score (per unit) | | 1.19 (1.12, 1.26) | 1.19 (1.12, 1.26) | 1.20 (1.11, 1.30) | 1.19 (1.10, 1.30) | 1.18 (1.09, 1.28) | 1.18 (1.09, 1.28) | 0.79 |

* MVPA Moderate-to-vigorous physical activity; Model 1 is adjusted for age and gender; Model 2 is additionally adjusted for BMI and IMD score
