# Supplementary material for: Associations between participation in organised physical activity in the school or community outside school hours and neighbourhood play with child physical activity and sedentary time: a cross-sectional analysis of primary school-aged children from the UK
Source: BMJ Open. 2017 Sep 14;7(9):e017588. doi: 10.1136/bmjopen-2017-017588 (PMC5640140; doi:10.1136/bmjopen-2017-017588)

**Figure S1 Scatter plot of sedentary time by activity score in the observed data (N=1003)**

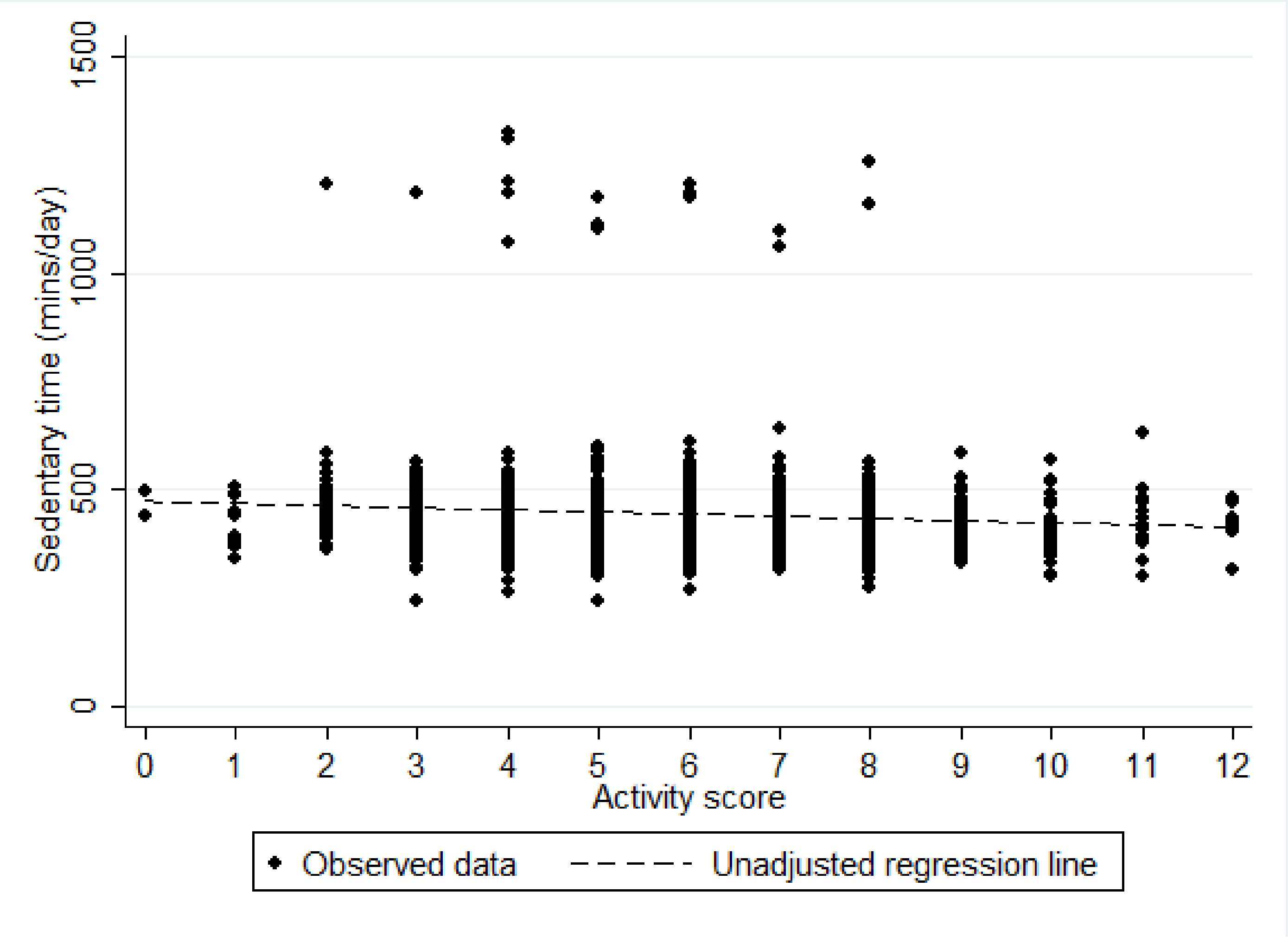

**Figure S2 Scatter plot of time spent in moderate-to-vigorous physical activity by activity score in the observed data (N=1003)**

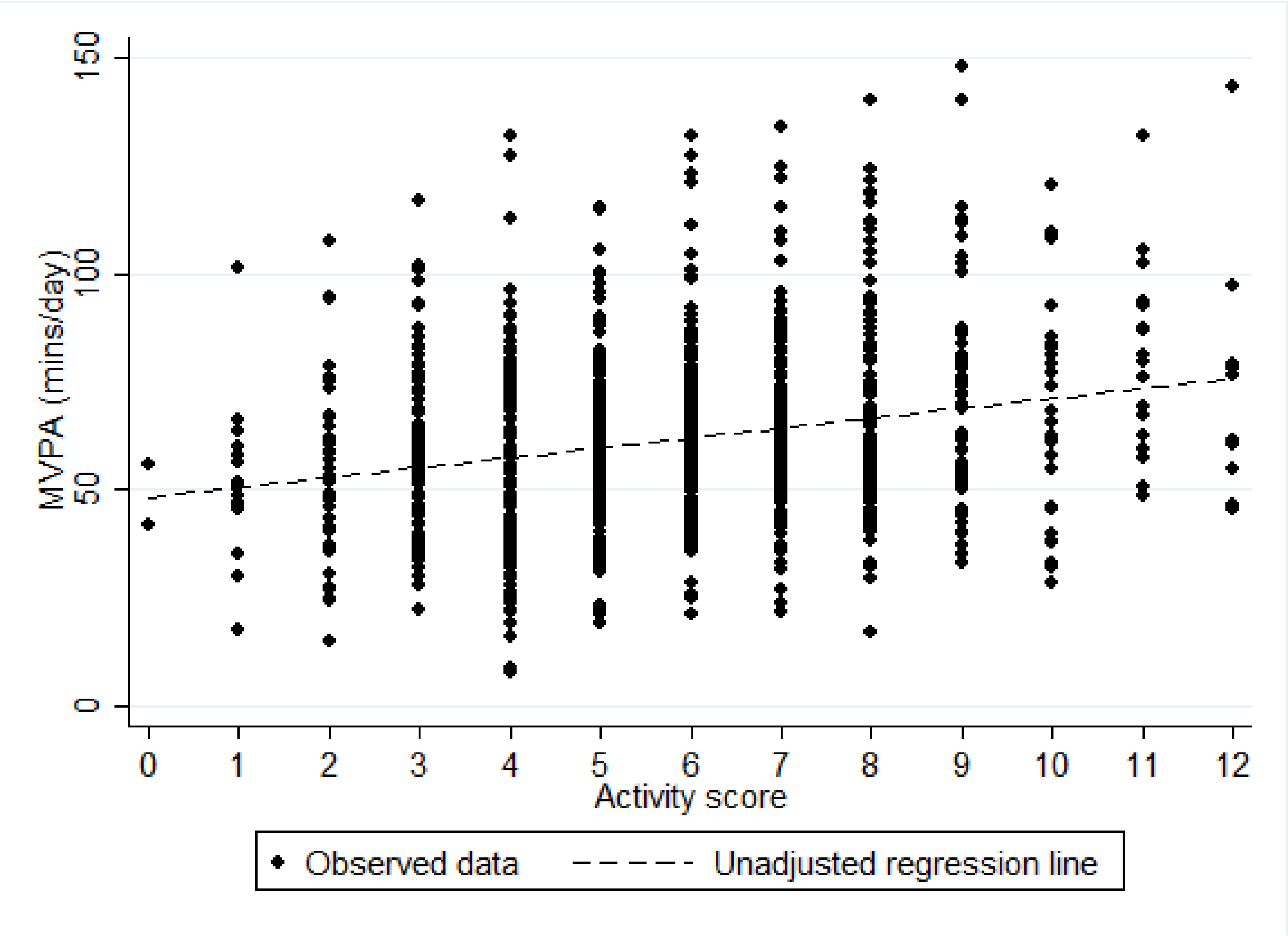

Supplement: Supplementary file 2 [file bmjopen-2017-017588supp002.pdf]
