# Supplementary material for: Associations between participation in organised physical activity in the school or community outside school hours and neighbourhood play with child physical activity and sedentary time: a cross-sectional analysis of primary school-aged children from the UK
Source: BMJ Open. 2017 Sep 14;7(9):e017588. doi: 10.1136/bmjopen-2017-017588 (PMC5640140; doi:10.1136/bmjopen-2017-017588)
Supplement: Author's manuscript [file bmjopen-2017-017588.draft_revisions.pdf]

BMJ Open is committed to open peer review. As part of this commitment we make the peer review history of every article we publish publicly available.

When an article is published we post the peer reviewers' comments and the authors' responses online. We also post the versions of the paper that were used during peer review. These are the versions that the peer review comments apply to.

The versions of the paper that follow are the versions that were submitted during the peer review process. They are not the versions of record or the final published versions. They should not be cited or distributed as the published version of this manuscript.

BMJ Open is an open access journal and the full, final, typeset and author-corrected version of record of the manuscript is available on our site with no access controls, subscription charges or pay-per-view fees (http://bmjopen.bmj.com).

If you have any questions on BMJ Open's open peer review process please email editorial.bmjopen@bmj.com

BMJ Open

Associations between participation in organised physical activity in the school or community outside school hours, and neighbourhood play with child physical activity and sedentary time: a cross-sectional analysis

Journal:	*BMJ Open*
Manuscript ID	bmjopen-2017-017588
Article Type:	Research
Date Submitted by the Author:	02-May-2017
Complete List of Authors:	Jago, Russell; Univ Bristol, Centre for Exercise, Nutrition & Health Sciences, School for Policy Studies<br>Macdonald-Wallis, Corrie; University of Bristol, Centre for Exercise, Nutrition & Health Sciences, School for Policy Studies, University of Bristol<br>Solomon-Moore, Emma; Univ Bristol, Centre for Exercise, Nutrition & Health Sciences, School for Policy Studies<br>Thompson, Janice; The University of Birmingham, School of Sport, Exercise and Rehabilitation Sciences<br>Lawlor, Debbie; University of Bristol School of Social and Community Medicine, MRC Integrative Epidemiology Unit at the University of Bristol<br>Sebire, Simon; University of Bristol, Centre for Exercise, Nutrition & Health Sciences, School for Policy Studies
**Primary Subject Heading**:	Public health
Secondary Subject Heading:	Paediatrics
Keywords:	Physical activity, children, accelerometer, extra-curricular, play

For peer review only

SCHOLARONE™
Manuscripts

Associations between participation in organised physical activity in the school or community outside school hours, and neighbourhood play with child physical activity and sedentary time: a cross-sectional analysis

Russell Jago [1$], Corrie Macdonald-Wallis [1], Emma Solomon-Moore[1], Janice L. Thompson[2], Deborah A. Lawlor [3,4] and Simon J. Sebire [1]

[1] Centre for Exercise, Nutrition & Health Sciences, School for Policy Studies, University of Bristol, 8 Priory Road, Bristol, BS8 1TZ, UK.

[2] School of Sport, Exercise and Rehabilitation Sciences, University of Birmingham, Birmingham, B15 2TT, UK.

[3] MRC Integrative Epidemiology Unit at the University of Bristol, Oakfield House, Oakfield Grove, Bristol, BS8 2BN, UK.

[4] School of Social and Community Medicine, University of Bristol, Canynge Hall, Whiteladies Road, Bristol, BS8 2PS, UK.

[$] **Corresponding author:** Prof Russell Jago, Centre for Exercise, Nutrition & Health Sciences, School for Policy Studies, University of Bristol, 8 Priory Road, Bristol, BS8 1TZ, UK. Email: russ.jago@bris.ac.uk; Telephone: +44 (0)117 9546603

Email addresses

RJ: russ.jago@bris.ac.uk

CMW: C.Macdonald-Wallis@bristol.ac.uk

ESM: Emma.solomon@bristol.ac.uk

JLT: j.thompson.1@bham.ac.uk

DAL: d.a.lawlor@bristol.ac.uk

SJS: simon.sebire@bristol.ac.uk

Word count: **Manuscript** = 2979 words **Abstract** = 298 words

For peer review only

ABSTRACT

Objectives: Assess the extent to which participation in organised physical activity in the school or community outside school hours, and neighbourhood play, were associated with children's physical activity and sedentary time.

Design: Cross-sectional study.

Setting: Children were recruited from 47 state funded primary schools in Southwest England.

Participants: 1223 8-9 year old children.

Outcome measures: Accelerometer-assessed moderate-to-vigorous-intensity physical activity (MVPA) and sedentary time.

Methods: Children wore an accelerometer and mean minutes of MVPA and sedentary time per day were derived. Children reported their attendance at organised physical activity in the school or community outside school hours, and neighbourhood play, using a piloted questionnaire. Cross-sectional linear and logistic regression were used to examine if attendance frequency at each setting (and all settings combined) was associated with moderate-to-vigorous-intensity physical activity (MVPA) and sedentary time. Multiple imputation methods were used to account for missing data and increase sample size.

Results: Children who attended clubs at school 3-4 days per week obtained an average of 7.58 (95% CI: 2.7 to 12.4) more minutes of MVPA per day than children who never attended. Report of participation in the three other non-school-based activities were similarly associated with MVPA. Evidence for associations with sedentary time was generally weaker. Associations were similar in girls and boys. When the four different contexts were combined, each additional 1-2 activities participated in per week increased participants' odds (OR: 1.18,

For peer review only - http://bmjopen.bmj.com/site/about/guidelines.xhtml

95% CI: 1.12 to 1.26) of meeting the Government recommendations for 60 minutes of MVPA per day.

Conclusion: Participating in organised physical activity at school and in the community is associated with greater physical activity and reduced sedentary time among both boys and girls. All four types of activity contribute to overall physical activity, which provides parents with a range of settings in which to help their child be active.

Key words: Physical activity, children, accelerometer, clubs, extra-curricular, play

For peer review only

For peer review only - http://bmjopen.bmj.com/site/about/guidelines.xhtml

ARTICLE SUMMARY

Strengths and limitations of this study

Strengths

- Accelerometer data from a large sample of Year 4 children.
- Detailed information on organised physical activity in the school or community outside school hours, and neighbourhood play.
- Multiple imputation models to provide estimates for participants with missing data.

Limitations

- Cross-sectional study design.
- Data are from a single UK region.

For peer review only - http://bmjopen.bmj.com/site/about/guidelines.xhtml

INTRODUCTION

Physical activity is associated with improved mental well-being, reduced risk of obesity and lower blood pressure among children.[1] Sedentary time may also be a risk factor for non-communicable diseases but it is not clear if this effect is independent of physical activity.[2-4] The UK Chief Medical Officers recommend that all children and young people should engage in at least an hour per day of moderate-to-vigorous-intensity physical activity (MVPA) and limit sedentary time,[5] however considerable proportions of children do not meet these guidelines.[6] For example, data from the nationally-representative Millennium cohort showed that only 51% of 7-8 year olds met the recommendation.[7] The amount of time children spend engaged in MVPA gradually declines with age, while sedentary time increases.[6 8-11] Strategies to increase children's physical activity are needed.

The majority of interventions to increase children's physical activity have been delivered during school time.[12 13] These interventions have included strategies such as changes to the physical education provision and new educational programmes based on information sharing and personal goal setting.[12-14] Overall, these programmes have tended to report no effect, weak effects or moderate effects in sub-groups.[12-16] Potential reasons for this are the difficulty faced in adding interventions to already full school-curricula and the lack of skills and training that teachers have for delivering a range of activities to engage the majority of children.[17] As such, there is a need to understand the potential of organised physical activity outside school hours to increase MVPA.

After-school programmes have the potential to facilitate physical activity for children, as schools have space in which children can be active, staff who can be trained, and many parents welcome programmes that provide childcare.[16 18-21] Although a number of studies

For peer review only - http://bmjopen.bmj.com/site/about/guidelines.xhtml

For peer review only

have examined the potential of delivering such sessions, it is not clear whether attendance at the programmes currently provided by schools is associated with higher overall levels of MVPA.[18-21] There is also a lack of information about how attendance at community-based physical activity clubs contributes to overall MVPA. Furthermore, as not all children attend after-school programmes, it is not clear how other activities such as playing in the neighbourhood or at home in the garden contribute to overall MVPA. A key question, therefore, is whether the frequency of participation in organised physical activity in the school or community after school hours, neighbourhood play or home play is associated with the MVPA and sedentary time of children. Since some children will be active in all four settings, it would also be informative to examine collective participation across all settings.

The aim of this study was to assess among children (8-9 years of age) the extent to which participation in organised physical activity in the school or community outside school hours, and playing with friends or family near the home or in the garden were associated with MVPA and sedentary time. A secondary aim was to examine if there was a cumulative association between participation in the four different types of activities with both MVPA and sedentary time.

METHODS

The current analyses used data from the B-Proact1v study, which has been described in detail elsewhere.[11 22 23] Briefly, the study aimed to examine physical activity behaviours of children and their parents over the course of primary school. Between 2012 and 2013, data were collected from 1299 Year 1 children (5-6 years of age) from 57 schools in Bristol (UK). Between March 2015 and July 2016, all 57 schools were approached to re-join the study when the children were in Year 4 (8-9 years of age), with 47 schools agreeing to take part

For peer review only - http://bmjopen.bmj.com/site/about/guidelines.xhtml

(1223 children). The current analyses used data from the Year 4 assessments. The study received ethical approval from the School for Policy Studies Ethics Committee at the University of Bristol, and written parent consent was received for all participants.

Data collection

Data were collected at schools, with children asked to complete a brief questionnaire. As indicators of organised physical activity outside school hours in school and in the community respectively, we asked "How often do you attend…a) a sport or exercise club at school (NOT including PE)? and b) a sport or exercise club at places other than your school (like a football club or ballet)?" To indicate neighbourhood play outside and within the home we asked "How much do you play with your friends and family… a) outside near your home? and b) in your home or garden?". These questions each had four response options: "Never", "1-2 days per week", "3-4 days per week" or "5 days per week". We assigned these 0, 1, 2 and 3 points respectively and summed responses to derive an overall activity score ranging from 0 to 12, with a higher value indicating a higher frequency of activity participation.

Child height was measured to the nearest 0.1cm using a SECA Leicester stadiometer (HAB International, Northampton). Weight was recorded to the nearest 0.1kg using a SECA 899 digital scale (HAB International, Northampton). Child Body Mass Index (BMI = kg/m^2) was then calculated and converted to an age- and gender-specific standard deviation score.[24 25] Children wore a waist-worn ActiGraph wGT3X-BT accelerometer for five days including two weekend days. Parents provided demographic information via a questionnaire, including child gender and date of birth. Where children's date of birth was missing (20.5% of children), they were assigned the median age of 9.0 years. Indices of Multiple Deprivation (IMD) scores, based upon the English Indices of Deprivation

For peer review only - http://bmjopen.bmj.com/site/about/guidelines.xhtml

(http://data.gov.uk/dataset/index-of-multiple-deprivation), were assigned to each child based on their reported home postcode, where higher IMD scores indicate a greater level of deprivation.

Accelerometer data reduction

Accelerometer data were processed using Kinesoft (v3.3.75; Kinesoft, Saskatchewan, Canada). At least three valid days of data were required for accelerometer data to be considered complete for a given child and included in analysis, where a valid day was defined as at least 500 minutes of data, after excluding intervals of ≥60 minutes of zero counts allowing up to two minutes of interruptions. The child's average number of sedentary and MVPA minutes per day were derived using population-specific cut points for children.[26] We also derived a binary variable indicating whether the child's average daily MVPA was greater than the 60 minutes per day recommended by the UK government.[5]

Analysis

The associations of child characteristics (gender, age, BMI z-score, and IMD) with activity participation were examined in the observed data using t-tests, Pearson's correlation coefficients, chi-squared tests and one-way analysis of variance as appropriate.

Multiple imputation of missing data was used to create 20 imputed datasets for the 1223 Year 4 children. We used 20 cycles of regression switching and combined regression coefficients across datasets using Rubin's rules.[27] We imputed separately for boys and girls to allow for associations to differ by gender. All exposures (organised physical activity attendance and neighbourhood play), outcomes (sedentary time and MVPA), potential confounders (gender, age, BMI z-score, and IMD) and child's school were included in multiple imputation models,

For peer review only - http://bmjopen.bmj.com/site/about/guidelines.xhtml

and achievement of the MVPA guideline and overall activity score were imputed passively. Any children with less than three valid days of accelerometer data had their accelerometer measures imputed.

We examined the pairwise associations of the activity participation variables by dichotomising, cross-tabulating and fitting unadjusted logistic regression models of one frequency variable on another.

We used linear regression models to examine the associations of activity participation and the overall activity score with the child's average sedentary and MVPA minutes per day, and logistic regression models to examine associations with achievement of the MVPA guideline. In Model 1 we adjusted for gender and age, and in Model 2 we adjusted additionally for BMI z-score and IMD. Robust standard errors were used to account for clustering of children within schools. Combined Wald tests were used to test for evidence of interaction between the child's gender and the exposure of interest.

We predicted the children's mean number of sedentary and MVPA minutes per day by frequency of participation in each activity based on linear combinations of the regression coefficients from fully-adjusted models (Model 2).

Regression analyses were repeated restricting to the children who had complete data for all exposures, outcomes and co-variables and compared with the multiple imputation analysis.

For peer review only - http://bmjopen.bmj.com/site/about/guidelines.xhtml

We also produced scatter plots of sedentary time and MVPA by the overall activity score in the observed data.

A sensitivity analysis was performed including accelerometer data for any children who had at least one valid day of measures to assess whether only including accelerometer data for children who recorded at least three valid days influenced our results. All analyses were performed in Stata version 14.0 (StataCorp, 2015).

RESULTS

The distributions of characteristics of the children in the observed data, multiple imputation datasets and subset with complete information are shown in Table 1. All characteristics showed similar distributions in each of the datasets and had only a small proportion of missing data (maximum 16.1% for accelerometer measures).

For peer review only

209 Table 1 Characteristics of children who took part in the Year 4 phase of the B-Proact1v Study (observed and multiple imputation) N=1223)

**Child Characteristic**		**Observed data**		**Imputed data** (N=1223)	**Complete data** (N=987)
		N available	Mean (SD) or %	Mean (SD) or %	Mean (SD) or %
Sedentary time at Year 4 (mins/day)		1026	445.4 (115.4)	444.7 (120.1)	446.0 (116.9)
MVPA at Year 4 (mins/day)		1026	61.6 (21.9)	61.9 (22.5)	61.8 (21.8)
Met MVPA guidelines at Year 4	No	1163	53.2	52.6	53.4
	Yes		46.8	47.4	46.6
Gender	Boy	1223	45.5	45.5	44.5
	Girl		54.5	54.5	55.5
Age at Year 4 (years)		1223	9.03 (0.41)	9.03 (0.41)	9.03 (0.43)
BMI z score at Year 4		1208	0.35 (1.08)	0.36 (1.08)	0.31 (1.07)
IMD score at Year 4		1204	15.9 (14.1)	15.9 (14.2)	15.3 (13.6)
Frequency child attends sport/exercise club at school	Never	1215	27.8	27.9	28.2
	1-2 days per week		45.5	45.5	45.8
	3-4 days per week		16.1	16.1	16.6
	5 days per week		10.5	10.5	9.4
Frequency child attends sport/exercise club outside school	Never	1214	20.6	20.6	19.4
	1-2 days per week		50.2	50.2	51.0
	3-4 days per week		20.8	20.9	21.5
	5 days per week		8.3	8.3	8.2
Frequency child plays with friends/family outside near home	Never	1205	6.3	6.4	6.5
	1-2 days per week		33.7	33.7	34.3
	3-4 days per week		29.0	28.9	29.7
	5 days per week		31.0	30.9	29.5
Frequency child plays with friends/family in home or garden	Never	1199	9.6	9.7	8.8
	1-2 days per week		34.5	34.6	35.0
	3-4 days per week		26.8	26.7	27.8
	5 days per week		29.1	29.0	28.5
Activity frequency score		1193	5.88 (2.29)	5.86 (2.29)	5.84 (2.26)

For peer review only - http://bmjopen.bmj.com/site/about/guidelines.xhtml

Associations of child characteristics with activity participation

Table S1 shows child activity attendance by gender. Girls tended to report lower frequencies of participating in organised physical activity in the school or community outside school hours, and had a lower mean overall activity score. There was no gender difference in friends/family play either in or outside of the home. The associations of other child characteristics with activity participation are shown in Table S2. There was some evidence that children who more frequently attended a sport/exercise club outside of school had a higher mean age. Child BMI was not strongly associated with any particular activity, but there was weak evidence that the overall activity score decreased with increasing BMI Z-score. Children who reported attendance of "Never" or "5 days/week" generally had the highest IMD scores, suggesting a U-shaped association, and there was a negative correlation between the overall activity score and IMD.

Inter-relationships of activity participation frequencies

Participating in one type of activity more frequently was generally associated with a higher frequency of participation in each of the others, except that attendance at a sport/exercise club outside of school was not associated with playing outside near the home (Table S3).

Associations of activity participation with sedentary time and MVPA

There was a negative correlation of the overall activity score with sedentary time and a positive correlation between the overall activity score and MVPA (Figures S1 and S2).

Table 2 shows the mean difference in sedentary time by activity participation and overall activity score in the multiple imputation datasets, and Figure 1 shows the predicted sedentary time by activity participation. Sedentary time decreased on average with increasing frequency

For peer review only - http://bmjopen.bmj.com/site/about/guidelines.xhtml

For peer review only

of attending sport/exercise clubs either at school or outside of school and with increasing frequency of playing with friends/family outside near the home in regression models adjusted for gender and age (Model 1). The association between sport/exercise club attendance outside of school and sedentary time weakened slightly on additional adjustment for BMI Z-score and IMD (Model 2) but other associations remained. An increase in children's overall activity score was also strongly associated with a reduction in sedentary time in both models. However, there was no evidence of an association between playing with friends/family at home and sedentary time. Associations did not differ between boys and girls. Findings were similar when restricted to children who had complete data (Table S4).

For peer review only

For peer review only - http://bmjopen.bmj.com/site/about/guidelines.xhtml

Table 2 Mean difference in the children's average sedentary minutes per day associated with different activities using multiple imputation (N=1223)*

Exposure		Sedentary time (minutes/day): mean difference (95% confidence interval)						
		All (N=1223)		Boys (N=556)		Girls (N=667)		P for gender interaction
		Model 1	Model 2	Model 1	Model 2	Model 1	Model 2	
Frequency child attends sport/exercise club at school	Never (ref)	0	0	0	0	0	0	0.69
	1-2 days/week	-4.2 (-22.5, 14.0)	-3.0 (-21.4, 15.4)	7.9 (-16.6, 32.3)	8.9 (-15.9, 33.7)	-13.0 (-38.3, 12.4)	-11.5 (-37.5, 14.4)	
	3-4 days/week	-31.1 (-50.7, -11.5)	-28.8 (-46.4, -11.3)	-18.7 (-41.8, 4.4)	-16.9 (-41.2, 7.3)	-40.1 (-71.7, -8.5)	-37.3 (-65.4, -9.2)	
	5 days/week	-18.7 (-42.9, 5.6)	-18.8 (-42.1, 4.5)	-9.6 (-37.7, 18.4)	-7.3 (-34.9, 20.4)	-26.0 (-66.8, 14.8)	-33.3 (-82.0, 15.5)	
P for trend		0.02	0.01	0.17	0.23	0.05	0.04	
Frequency child attends sport/exercise club outside of school	Never (ref)	0	0	0	0	0	0	0.81
	1-2 days/week	14.8 (-9.1, 38.7)	18.0 (-8.9, 45.0)	21.5 (-7.4, 50.5)	25.9 (-4.5, 56.4)	11.0 (-19.9, 42.0)	13.6 (-20.5, 47.7)	
	3-4 days/week	-7.2 (-27.0, 12.6)	-1.5 (-23.4, 20.4)	-1.0 (-32.8, 30.8)	4.8 (-27.1, 36.6)	-10.5 (-30.5, 9.5)	-4.1 (-27.3, 19.0)	
	5 days/week	-19.9 (-41.1, 1.2)	-15.5 (-37.7, 6.8)	-12.6 (-42.4, 17.1)	-7.7 (-36.5, 21.1)	-25.2 (-55.0, 4.6)	-20.6 (-52.1, 11.0)	
P for trend		0.02	0.07	0.12	0.16	0.07	0.23	
Frequency child plays with friends/family outside near home	Never (ref)	0	0	0	0	0	0	0.56
	1-2 days/week	0.77 (-28.5, 30.0)	2.3 (-28.0, 32.7)	19.2 (-16.5, 55.0)	21.2 (-14.4, 56.8)	-16.6 (-63.1, 30.0)	-15.6 (-65.0, 33.9)	
	3-4 days/week	-5.2 (-29.9, 19.6)	-2.8 (-29.4, 23.7)	7.6 (-28.4, 43.5)	10.6 (-25.5, 46.6)	-17.6 (-65.8, 30.5)	-16.1 (-67.4, 35.3)	
	5 days/week	-23.4 (-49.0, 2.1)	-24.1 (-50.8, 2.7)	-11.0 (-45.2, 23.2)	-10.2 (-44.4, 24.0)	-35.4 (-79.9, 9.0)	-37.8 (-86.6, 10.9)	
P for trend		0.004	0.001	0.07	0.05	0.02	0.02	
Frequency child plays with friends/family in home/garden	Never (ref)	0	0	0	0	0	0	0.90
	1-2 days/week	16.2 (-13.8, 46.3)	17.9 (-12.5, 48.2)	19.2 (-22.9, 61.3)	20.6 (-21.5, 62.7)	13.1 (-25.8, 52.0)	14.9 (-24.3, 54.1)	
	3-4 days/week	-3.9 (-28.8, 20.9)	-0.7 (-26.7, 25.4)	3.9 (-30.5, 38.4)	6.5 (-28.7, 41.7)	-10.7 (-46.6, 25.3)	-6.4 (-42.9, 30.1)	
	5 days/week	5.3 (-22.9, 33.5)	6.1 (-22.5, 34.7)	9.9 (-32.5, 52.3)	11.6 (-31.2, 54.4)	1.2 (-39.2, 41.5)	0.6 (-40.2, 41.5)	
P for trend		0.49	0.49	0.85	0.91	0.49	0.45	
Activity score (per unit)		-4.8 (-7.3, -2.3)	-4.6 (-7.0, -2.1)	-3.8 (-7.7, 0.1)	-3.4 (-7.2, 0.4)	-5.9 (-10.0, -1.7)	-5.9 (-10.2, -1.6)	0.41

* Model 1 is adjusted for age and gender; Model 2 is additionally adjusted for BMI and IMD score

For peer review only

For peer review only - http://bmjopen.bmj.com/site/about/guidelines.xhtml

The mean difference in MVPA by each of the activity variables in the multiple imputation data is shown in Table 3, with predicted MVPA by activity participation presented in Figure 2. A higher frequency of attending sport/exercise clubs either at school or outside of school and of play either outside or in the home/garden were all associated with greater MVPA on average in Models 1 and 2. Associations were similar in boys and girls. A higher overall activity score was also associated with greater MVPA, with some evidence that this association was stronger in boys than in girls. Associations were similar when restricted to children with complete data (Table S5).

For peer review only

For peer review only - http://bmjopen.bmj.com/site/about/guidelines.xhtml

Table 3 Mean difference in the children's average MVPA minutes per day associated with different activities using multiple imputation (N=1223)*

Exposure		Moderate-to-vigorous physical activity (minutes/day): mean difference (95% confidence interval)						
		All (N=1223)		Boys (N=556)		Girls (N=667)		P for gender interaction
		Model 1	Model 2	Model 1	Model 2	Model 1	Model 2	
Frequency child attends sport/exercise club at school	Never (ref)	0	0	0	0	0	0	0.64
	1-2 days/week	3.7 (0.3, 7.2)	3.6 (0.1, 7.0)	4.4 (-2.1, 10.9)	4.1 (-2.3, 10.5)	3.2 (-0.3, 6.7)	3.1 (-0.3, 6.6)	
	3-4 days/week	7.8 (2.9, 12.6)	7.5 (2.7, 12.4)	10.1 (2.6, 17.7)	9.6 (2.0, 17.2)	5.9 (0.9, 10.8)	5.8 (0.9, 10.7)	
	5 days/week	5.9 (-0.1, 11.8)	5.7 (-0.3, 11.7)	7.5 (-0.4, 15.4)	7.0 (-1.1, 15.0)	3.8 (-3.7, 11.3)	3.6 (-4.1, 11.4)	
P for trend		0.007	0.01	0.02	0.03	0.05	0.06	
Frequency child attends sport/exercise club outside of school	Never (ref)	0	0	0	0	0	0	0.80
	1-2 days/week	0.9 (-2.5, 4.3)	0.7 (-2.9, 4.4)	1.8 (-4.7, 8.3)	1.3 (-5.5, 8.2)	0.5 (-3.3, 4.4)	0.5 (-3.4, 4.5)	
	3-4 days/week	6.9 (2.4, 11.5)	6.5 (1.9, 11.2)	8.9 (1.0, 16.8)	8.1 (0.1, 16.0)	5.5 (0.9, 10.1)	5.6 (1.1, 10.2)	
	5 days/week	9.9 (3.8, 16.0)	9.6 (3.3, 15.9)	11.8 (3.4, 20.1)	11.2 (2.4, 19.9)	8.2 (0.7, 15.7)	8.2 (0.8, 15.7)	
P for trend		<0.001	<0.001	0.001	0.002	0.007	0.006	
Frequency child plays with friends/family outside near home	Never (ref)	0	0	0	0	0	0	0.19
	1-2 days/week	0.7 (-4.6, 6.0)	0.4 (-4.9, 5.6)	0.0 (-9.3, 9.2)	-0.5 (-9.9, 8.9)	1.4 (-6.0, 8.8)	1.3 (-6.2, 8.7)	
	3-4 days/week	5.1 (-0.9, 11.1)	4.8 (-1.1, 10.7)	6.1 (-4.1, 16.3)	5.7 (-4.5, 15.9)	4.4 (-3.5, 12.3)	4.3 (-3.7, 12.2)	
	5 days/week	9.6 (4.0, 15.3)	9.5 (3.9, 15.2)	13.0 (3.3, 22.8)	12.9 (3.0, 22.7)	6.7 (-0.7, 14.0)	6.6 (-0.9, 14.0)	
P for trend		<0.001	<0.001	<0.001	<0.001	0.005	0.006	
Frequency child plays with friends/family in home/garden	Never (ref)	0	0	0	0	0	0	0.38
	1-2 days/week	2.0 (-2.4, 6.4)	1.6 (-2.7, 6.0)	-0.1 (-8.4, 8.3)	-0.6 (-8.9, 7.7)	3.8 (-2.2, 9.8)	3.6 (-2.4, 9.7)	
	3-4 days/week	5.6 (0.9, 10.3)	5.3 (0.4, 10.2)	5.6 (-3.1, 14.3)	5.4 (-3.5, 14.4)	5.7 (-0.6, 11.9)	5.5 (-0.9, 12.0)	
	5 days/week	7.4 (3.0, 11.8)	7.1 (2.7, 11.6)	8.3 (0.1, 16.6)	7.7 (-0.7, 16.1)	6.6 (0.7, 12.5)	6.5 (0.6, 12.5)	

For peer review only

For peer review only - http://bmjopen.bmj.com/site/about/guidelines.xhtml

P for trend	<0.001	<0.001	0.004	0.006	0.02	0.02	
Activity score (per unit)	2.1 (1.4, 2.7)	2.0 (1.4, 2.7)	2.6 (1.7, 3.4)	2.5 (1.6, 3.4)	1.6 (0.8, 2.3)	1.6 (0.8, 2.3)	0.06

*MVPA Moderate-to-vigorous physical activity; Model 1 is adjusted for age and gender; Model 2 is additionally adjusted for BMI and IMD score

For peer review only - http://bmjopen.bmj.com/site/about/guidelines.xhtml

The associations of activity variables with achievement of the hour per day government guideline in the multiple imputation data are shown in Table 4. A higher frequency of participation in any of the activities, or an increase in the overall activity score, was associated with increased odds of meeting the government guideline in both models. Associations were similar in girls and boys. A unit increase in the activity score was associated with around an 18% increase in the odds of achieving 60 minutes of MVPA per day. Findings were unchanged when restricting to those with complete data (Table S6).

For peer review only

Table 4 Odds ratios for achieving 60 minutes of MVPA per day associated with different activities using multiple imputation (N=1223)*

Exposure		Meeting government guideline: odds ratio (95% confidence interval)						P for gender interaction
		All (N=1223)		Boys (N=556)		Girls (N=667)		
		Model 1	Model 2	Model 1	Model 2	Model 1	Model 2	
Frequency child attends sport/exercise club at school	Never (ref)	1	1	1	1	1	1	0.34
	1-2 days/week	1.33 (0.98, 1.81)	1.31 (0.97, 1.78)	1.10 (0.65, 1.86)	1.07 (0.63, 1.81)	1.54 (1.06, 2.23)	1.54 (1.07, 2.21)	
	3-4 days/week	1.87 (1.16, 3.01)	1.83 (1.14, 2.93)	2.22 (1.09, 4.53)	2.14 (1.04, 4.40)	1.63 (0.94, 2.85)	1.63 (0.94, 2.82)	
	5 days/week	1.69 (1.03, 2.78)	1.67 (1.02, 2.76)	1.61 (0.83, 3.09)	1.55 (0.79, 3.02)	1.76 (0.84, 3.69)	1.77 (0.84, 3.73)	
P for trend		0.01	0.01	0.03	0.05	0.05	0.05	
Frequency child attends sport/exercise club outside of school	Never (ref)	1	1	1	1	1	1	0.93
	1-2 days/week	1.14 (0.80, 1.64)	1.13 (0.77, 1.64)	1.21 (0.74, 1.98)	1.18 (0.70, 1.99)	1.11 (0.68, 1.80)	1.12 (0.69, 1.82)	
	3-4 days/week	1.82 (1.22, 2.72)	1.75 (1.16, 2.65)	2.07 (1.11, 3.87)	1.95 (1.03, 3.67)	1.66 (0.99, 2.78)	1.69 (1.01, 2.83)	
	5 days/week	2.70 (1.57, 4.62)	2.63 (1.51, 4.58)	3.24 (1.49, 7.07)	3.15 (1.41, 7.06)	2.30 (1.12, 4.72)	2.33 (1.14, 4.77)	
P for trend		<0.001	<0.001	<0.001	0.001	0.009	0.008	
Frequency child plays with friends/family outside near home	Never (ref)	1	1	1	1	1	1	0.74
	1-2 days/week	1.15 (0.70, 1.88)	1.12 (0.69, 1.82)	0.98 (0.46, 2.09)	0.94 (0.43, 2.06)	1.47 (0.62, 3.45)	1.47 (0.63, 3.43)	
	3-4 days/week	1.88 (1.12, 3.16)	1.84 (1.11, 3.06)	1.62 (0.73, 3.58)	1.59 (0.70, 3.58)	2.32 (0.91, 5.91)	2.32 (0.92, 5.88)	
	5 days/week	2.11 (1.26, 3.52)	2.10 (1.26, 3.49)	2.08 (0.92, 4.69)	2.07 (0.91, 4.74)	2.35 (0.93, 5.99)	2.36 (0.93, 6.02)	
P for trend		<0.001	<0.001	0.003	0.002	0.01	0.02	
Frequency child plays with friends/family in home/garden	Never (ref)	1	1	1	1	1	1	0.21
	1-2 days/week	1.27 (0.84, 1.91)	1.23 (0.81, 1.86)	0.94 (0.52, 1.72)	0.90 (0.49, 1.64)	1.75 (0.84, 3.64)	1.75 (0.84, 3.65)	
	3-4 days/week	1.51 (1.00, 2.28)	1.46 (0.96, 2.23)	1.29 (0.70, 2.40)	1.28 (0.68, 2.44)	1.80 (0.86, 3.80)	1.80 (0.86, 3.79)	
	5 days/week	1.71 (1.14, 2.56)	1.67 (1.11, 2.53)	1.71 (0.90, 3.25)	1.62 (0.83, 3.14)	1.80 (0.88, 3.69)	1.81 (0.88, 3.70)	

For peer review only - http://bmjopen.bmj.com/site/about/guidelines.xhtml

For peer review only

P for trend	0.005	0.006	0.01	0.02	0.21	0.21	
Activity score (per unit)	1.18 (1.12, 1.25)	1.18 (1.11, 1.25)	1.21 (1.12, 1.30)	1.20 (1.11, 1.30)	1.16 (1.07, 1.25)	1.16 (1.07, 1.25)	0.52

[*] MVPA Moderate-to-vigorous physical activity; Model 1 is adjusted for age and gender; Model 2 is additionally adjusted for BMI and IMD score

For peer review only

For peer review only - http://bmjopen.bmj.com/site/about/guidelines.xhtml

Sensitivity Analysis

When we changed the inclusion criteria for accelerometer measures from three to one valid day, findings were largely unchanged, except that boys showed a stronger association between sport/exercise club attendance at their school and MVPA than girls (p for interaction = 0.03 in multiple imputation data).

DISCUSSION

The findings demonstrate that increased participation in organised physical activity at school and in the community are associated with greater overall physical activity and reduced sedentary time among both boys and girls. Specifically, a child who attends a school-based club 3-4 days per week obtained 7.8 more minutes of MVPA per day than a child who did not attend at all, with attendance of five days a week at a sport/exercise club outside of school associated with 10.9 more minutes of MVPA than children who never attended clubs. There were comparable patterns for engagement in non-organised activity at home or in the neighbourhood, both were associated with increased MVPA, but only activity outside of the home was associated with reduced sedentary time. When the four different contexts of physical activity were combined, the analyses showed that each additional 1-2 activities participated in per week increased the odds of meeting the Chief Medical Officers' recommendation of 60 minutes of MVPA per day by 18%. Thus, encouraging children to attend after-school and community-based physical activity clubs, as well as to play at home and in the neighbourhood is critical for helping children to increase MVPA. Moreover, in light of the relative consistency in findings for each of the four forms of physical activity, the message to parents should be that physical activity can be accumulated in all four settings which allows them to find a balance that works for their family. For some families with working parents, after-school programmes may be the key activity to focus on, whereas for

For peer review only - http://bmjopen.bmj.com/site/about/guidelines.xhtml

other families encouraging children to play in the neighbourhood is likely to be useful for maximising physical activity. Furthermore, as there was little evidence that play at home was associated with a reduction in sedentary time, it is also important to examine ways of encouraging non-sedentary activities within the home.

The UK Childhood Obesity strategy recommends that all primary schools should provide at least 30 minutes per day of physical activity opportunities across the curriculum, break times and extra-curricular activities.[28] The data presented here show that in the imputed dataset 72.2% of Year 4 children were attending school-based programmes at least once per week and 10.5% were attending five days per week. Previous research has shown that in the UK, after-school clubs for primary school children tend to be dominated by team sports, such as football and rugby, with limited provision for non-competitive physical activities.[29] Thus, increasing the number and variety of sessions that children attend and improving the quality of those sessions is likely to provide a cost effective means of increasing children's physical activity. This hypothesis is consistent with the recent Theory of Expanded, Extended and Enhanced Opportunities (TEO), which suggests that the most effective means of increasing children's physical activity will be provided by extending and expanding current provision.[19] Thus, schools and community groups should be encouraged to extend current after-school provision to more children, diversify the activities to interest more pupils (preferably involving pupils in deciding what activities to offer), and enhance the quality of provision to maximise the amount of activity obtained. These relatively simple changes could be made at each school and would provide scalable ways for increasing overall levels of physical activity and contributing to the UK government's goal of reducing the prevalence of childhood obesity.

For peer review only

Strengths and limitations

The major strength of this study is the large sample size and provision of detailed information about participation in four different physical activity settings alongside accelerometer-assessed physical activity. In addition, the use of multiple imputation models to provide estimates for participants with missing data using a robust methodology has enabled us to maximise the sample for analysis. The study is limited by the cross-sectional design, which limits the ability to infer causation between frequency of participation in different settings and levels of physical activity. All questions were self-reported and it is possible that some were recalled more accurately than others. The report of play within the home is likely to include both sedentary and physically active forms of play, as the question included play indoors, which could be expected to be more sedentary, as well as outdoors in the garden, which is likely to be more active. We were unable to disentangle these. We also cannot rule out the possibility of residual confounding, but have adjusted for several key potential confounding variables in order to minimise this. The study is also drawn from the greater Bristol area in the UK and as such our ability to extend findings to other settings and countries is limited.

CONCLUSIONS

Participation in organised physical activity at school and in the community is associated with greater physical activity and reduced sedentary time among both boys and girls. In light of the challenges of promoting physical activity during school time, parents should encourage children to attend after school clubs, attend community activity groups and play in the neighbourhood to help their children to meet physical activity guidelines. The data show that all four types of activity contribute similarly to overall physical activity and there is therefore an opportunity for families to find the best mix of options for them.

For peer review only - http://bmjopen.bmj.com/site/about/guidelines.xhtml

ACKNOWLEDGEMENTS

This work was supported by grants from the British Heart Foundation (ref PG/11/51/28986 and SP 14/4/31123). DAL works in a unit that receives funding from the University of Bristol and UK Medical Research Council (MC_UU_1201/5); she is also a UK National Institute of Health and Research Senior Investigator (NF-SI-0166-10196). The funders had no involvement in data analysis, data interpretation or writing of the paper.

We would like to thank all of the families and schools that have taken part in the B-PROACT1V project. We would also like to thank all current and previous members of the research team who are not authors on this paper.

COMPETING INTERESTS

All authors have completed the ICMJE uniform disclosure form at www.icmje.org/coi_disclosure.pdf and declare: all authors had financial support from the British Heart Foundation for the submitted work; no financial relationships with any organisations that might have an interest in the submitted work in the previous three years; no other relationships or activities that could appear to have influenced the submitted work.

CONTRIBUTORS

Conception / design**:** RJ, ESM, JLT, DAL and SJS.

Data analysis / acquisition/ interpretation: RJ, CMW, ESM and DAL

Drafting / revising critically for important content: All authors.

Final approval: All authors.

Accountability for study and manuscript: RJ.

For peer review only - http://bmjopen.bmj.com/site/about/guidelines.xhtml

DATA SHARING STATEMENT: The datasets generated during the current study are not publicly available as the project is ongoing and data are not ready for archiving. We will consider reasonable requests for access to the data once the project is complete in 2019.

REFERENCES

1. Strong WB, Malina RM, Blimkie CJ, et al. Evidence based physical activity for school-age youth. J Pediatr 2005;**146**(6):732-7

2. Carson V, Hunter S, Kuzik N, et al. Systematic review of sedentary behaviour and health indicators in school-aged children and youth: an update. Applied physiology, nutrition, and metabolism = Physiologie appliquee, nutrition et metabolisme 2016;**41**(6 Suppl 3):S240-65 doi: 10.1139/apnm-2015-0630[published Online First: Epub Date]|.

3. Ekelund U, Luan J, Sherar LB, et al. Moderate to vigorous physical activity and sedentary time and cardiometabolic risk factors in children and adolescents. JAMA 2012;**307**(7):704-12 doi: 10.1001/jama.2012.156[published Online First: Epub Date]|.

4. Ekelund U, Steene-Johannessen J, Brown WJ, et al. Does physical activity attenuate, or even eliminate, the detrimental association of sitting time with mortality? A harmonised meta-analysis of data from more than 1 million men and women. Lancet 2016;**388**(10051):1302-10 doi: 10.1016/S0140-6736(16)30370-1[published Online First: Epub Date]|.

5. Department of Health PA, Health Improvement and Protection,. Start Active, Stay Active: A report on physical activity from the four home countries' Chief Medical Officers. London, 2011.

6. Cooper AR, Goodman A, Page AS, et al. Objectively measured physical activity and sedentary time in youth: the International children's accelerometry database (ICAD).

For peer review only - http://bmjopen.bmj.com/site/about/guidelines.xhtml

Int J Behav Nutr Phys Act 2015;**12**:113 doi: 10.1186/s12966-015-0274-5[published Online First: Epub Date]|.

7. Griffiths LJ, Cortina-Borja M, Sera F, et al. How active are our children? Findings from the Millennium Cohort Study. BMJ open 2013;**3**(8):e002893 doi: 10.1136/bmjopen-2013-002893[published Online First: Epub Date]|.

8. Nader PR, Bradley RH, Houts RM, McRitchie SL, O'Brien M. Moderate-to-vigorous physical activity from ages 9 to 15 years. JAMA 2008;**300**(3):295-305

9. Biddle SJ, Pearson N, Ross GM, Braithwaite R. Tracking of sedentary behaviours of young people: A systematic review. Prev Med 2010;**51**:345–51

10. Farooq MA, Parkinson KN, Adamson AJ, et al. Timing of the decline in physical activity in childhood and adolescence: Gateshead Millennium Cohort Study. Br J Sports Med 2017:1-6 doi: doi:10.1136/bjsports-2016-096933[published Online First: Epub Date]|.

11. Jago R, Solomon-Moore E, McDonald-Wallis C, Sebire SJ, Thompson JL, Lawlor DA. Change in children's physical activity and sedentary time between Year 1 and Year 4 of primary school in the B-PROACT1V cohort. Int J Behav Nutr Phys Act In Press

12. Metcalf B, Henley W, Wilkin T. Effectiveness of intervention on physical activity of children: systematic review and meta-analysis of controlled trials with objectively measured outcomes (EarlyBird 54). BMJ 2012;**345**:e5888 doi: 10.1136/bmj.e5888[published Online First: Epub Date]|.

13. van Sluijs EM, McMinn AM, Griffin SJ. Effectiveness of interventions to promote physical activity in children and adolescents: systematic review of controlled trials. BMJ 2007;**335**(7622):703

14. Kipping R, Payne C, Lawlor DA. Randomised controlled trial adapting American school obesity prevention to England. Arch Dis Child 2008

For peer review only

For peer review only - http://bmjopen.bmj.com/site/about/guidelines.xhtml

15. McGoey T, Root Z, Bruner MW, Law B. Evaluation of physical activity interventions in children via the reach, efficacy/effectiveness, adoption, implementation, and maintenance (RE-AIM) framework: A systematic review of randomized and non-randomized trials. Prev Med 2016;**82**:8-19 doi: 10.1016/j.ypmed.2015.11.004[published Online First: Epub Date]|.

16. Arundell L, Fletcher E, Salmon J, Veitch J, Hinkley T. A systematic review of the prevalence of sedentary behavior during the after-school period among children aged 5-18 years. Int J Behav Nutr Phys Act 2016;**13**:93 doi: 10.1186/s12966-016-0419-1[published Online First: Epub Date]|.

17. Jago R, Rawlins E, Kipping RR, et al. Lessons learned from the AFLY5 RCT process evaluation: implications for the design of physical activity and nutrition interventions in schools. BMC Public Health 2015;**15**:946 doi: 10.1186/s12889-015-2293-1[published Online First: Epub Date]|.

18. Beets MW, Beighle A, Erwin HE, Huberty JL. After-school program impact on physical activity and fitness: a meta-analysis. Am J Prev Med 2009;**36**(6):527-37 doi: 10.1016/j.amepre.2009.01.033[published Online First: Epub Date]|.

19. Beets MW, Okely A, Weaver RG, et al. The theory of expanded, extended, and enhanced opportunities for youth physical activity promotion. Int J Behav Nutr Phys Act 2016;**13**(1):120 doi: 10.1186/s12966-016-0442-2[published Online First: Epub Date]|.

20. Beets MW, Weaver RG, Turner-McGrievy G, et al. Physical activity outcomes in afterschool programs: A group randomized controlled trial. Prev Med 2016;**90**:207-15 doi: 10.1016/j.ypmed.2016.07.002[published Online First: Epub Date]|.

21. Weaver RG, Beets MW, Huberty J, Freedman D, Turner-Mcgrievy G, Ward D. Physical activity opportunities in afterschool programs. Health promotion practice

For peer review only

For peer review only - http://bmjopen.bmj.com/site/about/guidelines.xhtml

2015;**16**(3):371-82 doi: 10.1177/1524839914567740[published Online First: Epub Date]|.

22. Jago R, Sebire SJ, Wood L, et al. Associations between objectively assessed child and parental physical activity: a cross-sectional study of families with 5-6 year old children. BMC Public Health 2014;**14**:655 doi: 10.1186/1471-2458-14-655[published Online First: Epub Date]|.

23. Jago R, Thompson JL, Sebire SJ, et al. Cross-sectional associations between the screen-time of parents and young children: differences by parent and child gender and day of the week. Int J Behav Nutr Phys Act 2014;**11**:54 doi: 10.1186/1479-5868-11-54[published Online First: Epub Date]|.

24. Cole TJ, Bellizzi MC, Flegal KM, Dietz WH. Establishing a standard definition for child overweight and obesity worldwide: international survey. BMJ 2000;**320**(7244):1240-3

25. Cole TJ, Freeman JV, Preece MA. Body mass index reference curves for the UK, 1990. Arch Dis Child 1995;**73**(1):25-9

26. Evenson KR, Catellier DJ, Gill K, Ondrak KS, McMurray RG. Calibration of two objective measures of physical activity for children. Journal of sports sciences 2008;**26**(14):1557-65

27. Rubin DB. Multiple imputation after 18+ years. Journal of the American Statistical Association 1996;**91**(434):473-789

28. Cabinet Office. Childhood obesity: a plan for action. In: Prime Minister's Office DS, ed. London: Prime Minister's Office, 10 Downing Street, 2017.

29. Davies B, Wood L, Banfield K, Edwards MJ, Jago R. The provision of active after-school clubs for children in English primary schools: implications for increasing children's physical activity. Open Journal of Preventive Medicine 2014;**4**:598-605 doi: http://dx.doi.org/10.4236/ojpm.2014.47069[published Online First: Epub Date]|.

For peer review only

For peer review only - http://bmjopen.bmj.com/site/about/guidelines.xhtml

Figure 1 Predicted time spent in sedentary behaviour by type of activity using multiple imputation (N=1223)*

Figure 2 Predicted time spent in moderate-to-vigorous physical activity by type of activity using multiple imputation (N=1223)*

For peer review only

For peer review only - http://bmjopen.bmj.com/site/about/guidelines.xhtml

For peer review only

For peer review only - http://bmjopen.bmj.com/site/about/guidelines.xhtml

Figure 1 Predicted time spent in sedentary behaviour by type of activity using multiple imputation (N=1223)*

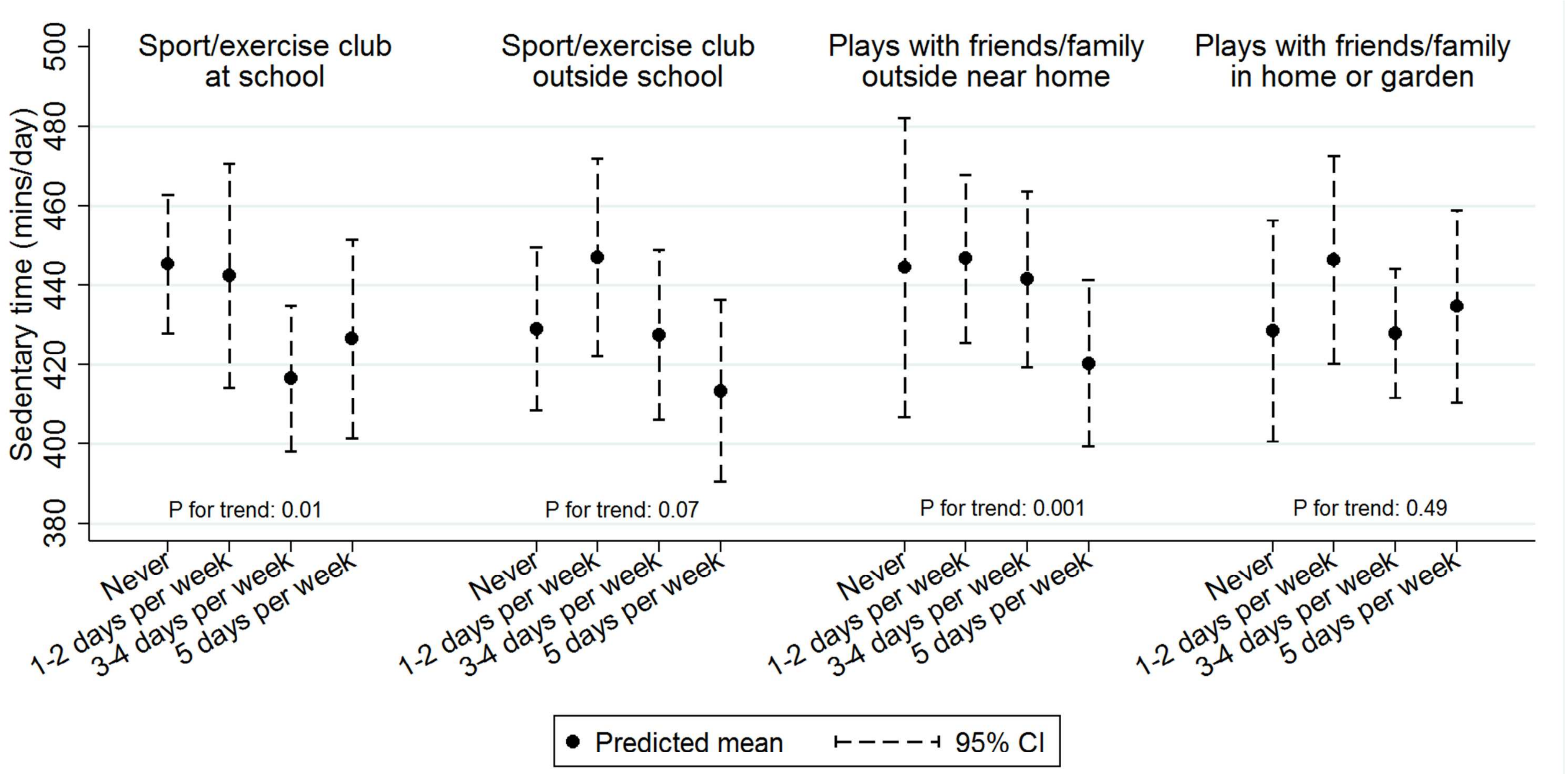

* Predictions were obtained from fully adjusted regression models (Model 2) including all Year 4 children (boys and girls) and are for a 9-year old boy with a BMI z-score of 0 and IMD score of 16. Predicted sedentary time for girls was approximately 15-17 mins/day higher (same additive effect across all categories of the exposure variable) depending on the regression model

Figure 1 Predicted time spent in moderate-to-vigorous physical activity by type of activity using multiple imputation (N=1223)*

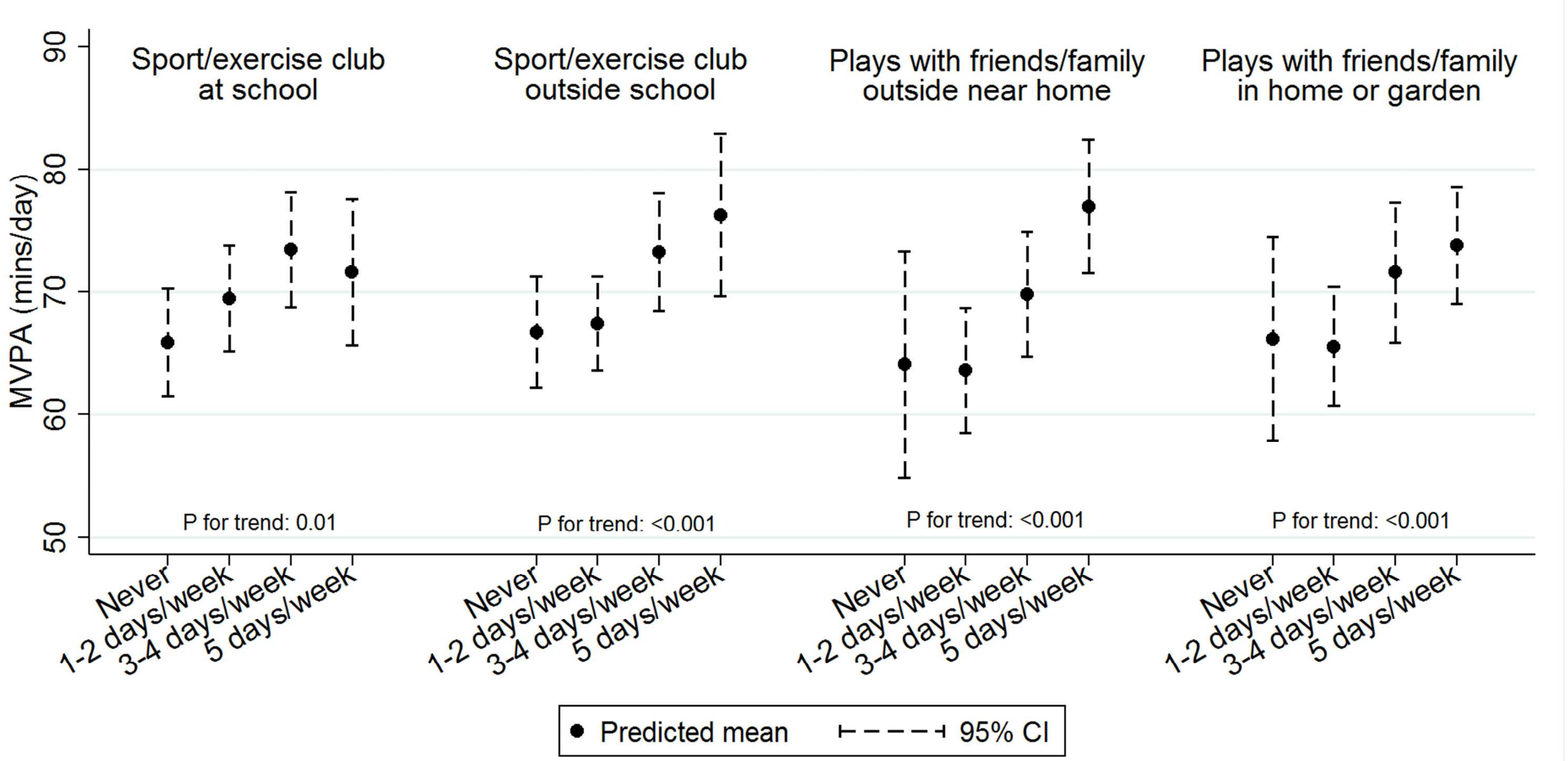

[*] Predictions were obtained from fully adjusted regression models (Model 2) including all Year 4 children (boys and girls) and are for a 9-year old boy with a BMI z-score of 0 and IMD score of 16. Predicted time spent in moderate-to-vigorous physical activity for girls was approximately 13 mins/day lower (same additive effect across all categories of the exposure variable)

Table S1 Frequencies of sport/exercise club attendance and play outside and inside the home and mean activity score by gender in the observed data

		Boys<br>N (%)	Girls<br>N (%)	Chi-squared p-value for association
**Frequency child attends sport/exercise club at school**	Never<br>1-2 days/week<br>3-4 days/week<br>5 days/week	144 (25.9)<br>235 (42.3)<br>90 (16.2)<br>86 (15.5)	194 (29.4)<br>318 (48.2)<br>106 (16.1)<br>42 (6.4)	<0.001
**Frequency child attends sport/exercise club outside of school**	Never<br>1-2 days/week<br>3-4 days/week<br>5 days/week	85 (15.3)<br>289 (52.1)<br>121 (21.8)<br>60 (10.8)	165 (25.0)<br>321 (48.7)<br>132 (20.0)<br>41 (6.2)	<0.001
**Frequency child plays with friends/family outside near home**	Never<br>1-2 days/week<br>3-4 days/week<br>5 days/week	36 (6.6)<br>187 (34.1)<br>142 (25.9)<br>184 (33.5)	40 (6.1)<br>219 (33.4)<br>207 (31.6)<br>190 (29.0)	0.14
**Frequency child plays with friends/family in home/garden**	Never<br>1-2 days/week<br>3-4 days/week<br>5 days/week	53 (9.7)<br>184 (33.7)<br>142 (26.0)<br>167 (30.6)	62 (9.5)<br>230 (35.2)<br>179 (27.4)<br>182 (27.9)	0.76

	Boys		Girls		T-test p-value for gender difference
	N	Mean (SD)	N	Mean (SD)	
**Activity frequency score**	542	6.15 (2.42)	651	5.65 (2.16)	<0.001

For peer review only

		Age (years)		BMI z score		IMD score	
		Mean (SD)	N	Mean (SD)	N	Mean (SD)	N
**Frequency child attends sport/exercise club at school**	Never<br>1-2 days/week<br>3-4 days/week<br>5 days/week	9.03 (0.38)<br>9.05 (0.43)<br>9.00 (0.45)<br>8.99 (0.39)	1215	0.43 (1.14)<br>0.35 (1.05)<br>0.29 (1.02)<br>0.27 (1.13)	1202	17.30 (15.04)<br>15.54 (14.02)<br>14.15 (11.07)<br>16.66 (15.10)	1197
P-value for difference between categories*		0.31		0.36		0.07	
**Frequency child attends sport/exercise club outside of school**	Never<br>1-2 days/week<br>3-4 days/week<br>5 days/week	9.01 (0.40)<br>9.01 (0.43)<br>9.05 (0.41)<br>9.12 (0.39)	1214	0.47 (1.07)<br>0.37 (1.09)<br>0.23 (1.07)<br>0.30 (1.06)	1201	20.03 (16.06)<br>16.02 (14.12)<br>12.30 (10.25)<br>14.26 (13.64)	1196
P-value for difference between categories*		0.05		0.09		<0.001	
**Frequency child plays with friends/family outside near home**	Never<br>1-2 days/week<br>3-4 days/week<br>5 days/week	8.96 (0.47)<br>9.01 (0.42)<br>9.05 (0.41)<br>9.05 (0.40)	1205	0.48 (1.24)<br>0.31 (1.06)<br>0.39 (1.07)<br>0.33 (1.08)	1192	17.60 (16.02)<br>15.20 (13.83)<br>14.66 (12.48)<br>17.66 (15.08)	1187
P-value for difference between categories*		0.23		0.58		0.01	
**Frequency child plays with friends/family in home/garden**	Never<br>1-2 days/week<br>3-4 days/week<br>5 days/week	8.97 (0.38)<br>9.02 (0.45)<br>9.06 (0.41)<br>9.03 (0.39)	1199	0.50 (1.11)<br>0.29 (1.09)<br>0.40 (1.07)<br>0.32 (1.07)	1186	18.54 (15.94)<br>15.85 (13.95)<br>14.24 (12.79)<br>16.85 (14.55)	1181
P-value for difference between categories*		0.31		0.23		0.02	
		Correlation	P-value	Correlation	P-value	Correlation	P-value
**Activity frequency score**		0.04	0.13	-0.05	0.07	-0.07	0.02

For peer review only

Table S2 Child characteristics by frequencies of sport/exercise club attendance and play outside and inside the home and activity score in the observed data

For peer review only

Table S3 Pairwise comparisons of children's activity attendance frequencies in the multiple imputation data (N=1223)*

		Frequency child attends sport/exercise club at school (%)		Frequency child attends sport/exercise club outside of school (%)		Frequency child plays with friends/family outside near home (%)		Frequency child plays with friends/family in home/garden (%)	
		Up to 2 days/week	3 or more days/week	Up to 2 days/week	3 or more days/week	Up to 2 days/week	3 or more days/week	Up to 2 days/week	3 or more days/week
**Frequency child attends sport/exercise club at school**	Up to 2 days/week	[redacted]	[redacted]	76.3	23.7	42.7	57.3	47.3	52.7
	3 or more days/week	[redacted]	[redacted]	55.8	44.2	33.2	66.8	36.0	64.0
P for association		[redacted]		<0.001		0.003		0.001	
**Frequency child attends sport/exercise club outside of school**	Up to 2 days/week	79.0	21.0	[redacted]	[redacted]	40.2	59.8	46.8	53.2
	3 or more days/week	59.7	40.3	[redacted]	[redacted]	40.0	60.0	38.2	61.8
P for association		<0.001		[redacted]		0.94		0.006	
**Frequency child plays with friends/family outside near home**	Up to 2 days/week	78.0	22.0	71.0	29.0	[redacted]	[redacted]	65.1	34.9
	3 or more days/week	70.3	29.7	70.8	29.2	[redacted]	[redacted]	30.3	69.7
P for association		0.003		0.94		[redacted]		<0.001	
**Frequency child plays with friends/family in home/garden**	Up to 2 days/week	78.3	21.7	74.9	25.1	59.0	41.0	[redacted]	[redacted]
	3 or more days/week	69.4	30.6	67.6	32.4	25.2	74.8	[redacted]	[redacted]
P for association		0.001		0.006		<0.001		[redacted]	
**Total**		**73.4**	**26.6**	**70.8**	**29.2**	**40.2**	**59.8**	**44.3**	**55.7**

* Percentages presented are the proportions of children in each row that belong to each of the categories of child activity variables listed along the top of the table.

For peer review only

Table S4 Mean difference in the children's average sedentary minutes per day associated with different activities for those with complete data (N=987)*

Exposure		Sedentary time (minutes/day): mean difference (95% confidence interval)						
		All (N=987)		Boys (N=439)		Girls (N=548)		P for gender interaction
		Model 1	Model 2	Model 1	Model 2	Model 1	Model 2	
Frequency child attends sport/exercise club at school	Never (ref)	0	0	0	0	0	0	0.45
	1-2 days/week	-0.29 (-21.3, 20.7)	0.0 (-21.2, 21.3)	14.7 (-10.1, 39.5)	15.9 (-9.2, 41.0)	-11.4 (-39.7, 16.8)	-11.9 (-41.1, 17.3)	
	3-4 days/week	-26.3 (-40.8, -11.8)	-24.1 (-37.8, -10.4)	-13.8 (-31.5, 4.0)	-9.9 (-29.3, 9.4)	-36.0 (-59.5, -12.4)	-35.4 (-59.0, -11.7)	
	5 days/week	-16.0 (-35.3, 3.3)	-16.1 (-34.4, 2.3)	-7.1 (-29.7, 15.4)	-3.1 (-26.9, 20.6)	-22.0 (-54.6, 10.7)	-29.8 (-69.2, 9.66)	
P for trend		0.01	0.009	0.19	0.40	0.02	0.02	
Frequency child attends sport/exercise club outside of school	Never (ref)	0	0	0	0	0	0	0.73
	1-2 days/week	16.1 (-7.5, 39.7)	20.0 (-7.0, 47.0)	24.6 (-3.4, 52.7)	31.2 (0.6, 61.8)	10.8 (-19.3, 41.0)	12.9 (-20.0, 45.9)	
	3-4 days/week	-6.6 (-23.2, 10.1)	0.1 (-19.6, 19.8)	-3.2 (-28.8, 22.3)	5.6 (-17.7, 28.8)	-7.6 (-26.3, 11.1)	-2.0 (-26.7, 22.6)	
	5 days/week	-18.4 (-36.5, -0.2)	-13.8 (-33.8, 6.2)	-13.0 (-36.3, 10.4)	-5.9 (-28.8, 16.9)	-20.8 (-46.0, 4.3)	-18.2 (-46.4, 10.0)	
P for trend		0.009	0.05	0.02	0.03	0.08	0.28	
Frequency child plays with friends/family outside near home	Never (ref)	0	0	0	0	0	0	0.68
	1-2 days/week	4.5 (-26.7, 35.7)	6.0 (-27.5, 39.5)	16.3 (-13.4, 45.9)	20.2 (-10.1, 50.4)	-5.7 (-55.7, 44.3)	-7.1 (-59.0, 44.9)	
	3-4 days/week	-2.5 (-30.1, 25.0)	-0.6 (-31.6, 30.3)	8.3 (-23.1, 39.7)	13.0 (-19.4, 45.4)	-11.9 (-68.1, 44.3)	-13.2 (-71.3, 44.9)	
	5 days/week	-21.6 (-49.0, 5.7)	-21.9 (-50.4, 6.6)	-9.4 (-35.4, 16.6)	-6.4 (-32.8, 20.0)	-32.3 (-78.9, 14.2)	-36.4 (-86.0, 13.1)	
P for trend		<0.001	<0.001	0.08	0.08	0.009	0.01	
Frequency child plays with friends/family in home/garden	Never (ref)	0	0	0	0	0	0	0.86
	1-2 days/week	18.2 (-12.4, 48.8)	18.8 (-12.4, 50.0)	19.2 (-13.8, 52.2)	19.5 (-13.7, 52.8)	17.2 (-25.4, 59.9)	17.6 (-25.4, 60.6)	
	3-4 days/week	-4.5 (-27.3, 18.2)	-1.9 (-26.7, 22.9)	2.8 (-16.3, 21.9)	4.6 (-15.7, 25.0)	-10.1 (-47.3, 27.0)	-7.5 (-47.1, 32.1)	
	5 days/week	3.8 (-24.6, 32.2)	3.6 (-24.6, 31.8)	10.8 (-27.1, 48.8)	11.6 (-27.0, 50.2)	-2.2 (-43.0, 38.6)	-3.5 (-45.3, 38.3)	
P for trend		0.24	0.22	0.85	0.89	0.25	0.24	
Activity score (per unit)		-4.9 (-7.2, -2.7)	-4.7 (-6.9, -2.4)	-3.5 (-6.6, -0.4)	-2.9 (-6.0, 0.2)	-6.3 (-10.7, -2.0)	-6.6 (-11.2, -1.9)	0.28

For peer review only

* Model 1 is adjusted for age and gender; Model 2 is additionally adjusted for BMI and IMD score

For peer review only

Table S5 Mean difference in the children's average MVPA minutes per day associated with different activities for those with complete data (N=987)*

Exposure		Moderate-to-vigorous physical activity (minutes/day): mean difference (95% confidence interval)						
		All (N=987)		Boys (N=439)		Girls (N=548)		P for gender interaction
		Model 1	Model 2	Model 1	Model 2	Model 1	Model 2	
Frequency child attends sport/exercise club at school	Never (ref)	0	0	0	0	0	0	0.64
	1-2 days/week	3.2 (-0.4, 6.8)	3.1 (-0.5, 6.6)	3.3 (-3.3, 9.8)	2.7 (-3.7, 9.2)	3.1 (-0.5, 6.8)	3.1 (-0.6, 6.8)	
	3-4 days/week	6.7 (1.7, 11.8)	6.4 (1.4, 11.4)	8.0 (1.0, 15.1)	7.3 (0.1, 14.4)	5.7 (0.6, 10.7)	5.6 (0.5, 10.7)	
	5 days/week	5.3 (-1.3, 11.9)	5.1 (-1.5, 11.8)	6.6 (-1.4, 14.6)	5.8 (-2.2, 13.8)	3.2 (-5.0, 11.5)	3.1 (-5.5, 11.6)	
P for trend		0.02	0.03	0.02	0.05	0.08	0.10	
Frequency child attends sport/exercise club outside of school	Never (ref)	0	0	0	0	0	0	0.53
	1-2 days/week	1.8 (-1.9, 5.5)	1.5 (-2.4, 5.4)	4.3 (-3.2, 11.9)	3.6 (-4.4, 11.7)	0.4 (-3.5, 4.3)	0.4 (-3.5, 4.4)	
	3-4 days/week	7.3 (3.0, 11.5)	6.7 (2.4, 11.0)	10.5 (2.8, 18.2)	9.0 (1.4, 16.6)	5.3 (0.6, 10.0)	5.5 (0.9, 10.0)	
	5 days/week	10.5 (4.6, 16.5)	10.1 (3.9, 16.2)	14.3 (6.3, 22.3)	13.4 (4.8, 22.0)	7.3 (-0.8, 15.3)	7.3 (-0.6, 15.2)	
P for trend		<0.001	<0.001	<0.001	<0.001	0.01	0.008	
Frequency child plays with friends/family outside near home	Never (ref)	0	0	0	0	0	0	0.32
	1-2 days/week	0.7 (-4.1, 5.5)	0.4 (-4.3, 5.0)	1.2 (-7.4, 9.7)	0.5 (-8.1, 9.1)	0.4 (-6.0, 6.9)	0.3 (-6.3, 6.9)	
	3-4 days/week	5.2 (-0.8, 11.2)	4.9 (-1.0, 10.7)	6.7 (-3.5, 16.9)	6.0 (-4.0, 15.9)	4.0 (-3.6, 11.7)	3.9 (-3.8, 11.7)	
	5 days/week	9.9 (4.4, 15.4)	9.7 (4.2, 15.1)	13.8 (4.6, 23.0)	13.3 (4.0, 22.5)	6.5 (-0.5, 13.5)	6.4 (-0.7, 13.6)	
P for trend		<0.001	<0.001	<0.001	<0.001	0.004	0.005	
Frequency child plays with friends/family in home/garden	Never (ref)	0	0	0	0	0	0	0.40
	1-2 days/week	2.5 (-0.9, 6.0)	2.2 (-1.4, 5.8)	0.6 (-7.0, 8.2)	0.4 (-7.4, 8.2)	4.1 (-0.9, 9.2)	4.0 (-1.1, 9.1)	
	3-4 days/week	6.6 (2.4, 10.7)	6.3 (1.8, 10.8)	6.7 (-1.8, 15.3)	6.9 (-1.9, 15.7)	6.5 (0.4, 12.6)	6.4 (0.1, 12.7)	
	5 days/week	8.6 (4.6, 12.5)	8.4 (4.3, 12.6)	9.3 (1.6, 16.9)	9.1 (1.0, 17.2)	7.9 (2.5, 13.3)	7.8 (2.4, 13.3)	
P for trend		<0.001	<0.001	0.001	0.001	0.003	0.003	
Activity score (per unit)		2.13 (1.44, 2.82)	2.09 (1.37, 2.81)	2.58 (1.73, 3.43)	2.49 (1.55, 3.42)	1.67 (0.86, 2.47)	1.66 (0.85, 2.48)	0.07

For peer review only

For peer review only - http://bmjopen.bmj.com/site/about/guidelines.xhtml

Exposure		Meeting government guideline: odds ratio (95% confidence interval)						P for gender interaction
		All (N=987)		Boys (N=439)		Girls (N=548)		
		Model 1	Model 2	Model 1	Model 2	Model 1	Model 2	
Frequency child attends sport/exercise club at school	Never (ref)	1	1	1	1	1	1	0.25
	1-2 days/week	1.27 (0.94, 1.71)	1.25 (0.93, 1.69)	0.97 (0.58, 1.63)	0.93 (0.55, 1.55)	1.57 (1.09, 2.25)	1.57 (1.09, 2.26)	
	3-4 days/week	1.77 (1.05, 2.97)	1.72 (1.03, 2.87)	1.97 (0.93, 4.18)	1.86 (0.88, 3.95)	1.65 (0.93, 2.91)	1.66 (0.94, 2.92)	
	5 days/week	1.63 (0.96, 2.77)	1.61 (0.95, 2.74)	1.49 (0.75, 2.96)	1.40 (0.70, 2.77)	1.73 (0.78, 3.85)	1.76 (0.78, 3.96)	
P for trend		0.03	0.03	0.06	0.10	0.07	0.07	
Frequency child attends sport/exercise club outside of school	Never (ref)	1	1	1	1	1	1	0.66
	1-2 days/week	1.32 (0.91, 1.91)	1.29 (0.88, 1.88)	1.53 (0.89, 2.62)	1.46 (0.83, 2.57)	1.19 (0.72, 1.97)	1.21 (0.74, 1.99)	
	3-4 days/week	2.06 (1.38, 3.07)	1.97 (1.32, 2.95)	2.56 (1.32, 4.98)	2.28 (1.18, 4.40)	1.77 (1.05, 2.98)	1.81 (1.09, 3.03)	
	5 days/week	2.89 (1.76, 4.73)	2.80 (1.69, 4.63)	4.13 (1.97, 8.68)	3.92 (1.82, 8.44)	2.09 (1.06, 4.12)	2.14 (1.10, 4.18)	
P for trend		<0.001	<0.001	<0.001	<0.001	0.006	0.004	
Frequency child plays with friends/family	Never (ref)	1	1	1	1	1	1	0.89
	1-2 days/week	1.16 (0.70, 1.92)	1.13 (0.70, 1.85)	1.03 (0.46, 2.29)	0.98 (0.43, 2.22)	1.40 (0.59, 3.30)	1.41 (0.60, 3.29)	

* MVPA Moderate-to-vigorous physical activity; Model 1 is adjusted for age and gender; Model 2 is additionally adjusted for BMI and IMD score

For peer review only

Table S6 Odds ratios for achieving 60 minutes of MVPA per day associated with different activities for those with complete data (N=987)*

outside near home	3-4 days/week	1.94 (1.12, 3.35)	1.89 (1.12, 3.21)	1.59 (0.68, 3.75)	1.53 (0.65, 3.62)	2.43 (0.92, 6.45)	2.46 (0.94, 6.42)	
	5 days/week	2.14 (1.26, 3.65)	2.12 (1.26, 3.57)	2.01 (0.88, 4.63)	1.97 (0.85, 4.54)	2.45 (0.95, 6.36)	2.49 (0.97, 6.40)	
P for trend		<0.001	<0.001	0.007	0.005	0.007	0.006	
Frequency child plays with friends/family in home/garden	Never (ref)	1	1	1	1	1	1	0.30
	1-2 days/week	1.33 (0.88, 2.00)	1.30 (0.86, 1.97)	0.96 (0.51, 1.78)	0.94 (0.50, 1.77)	1.92 (0.90, 4.11)	1.94 (0.91, 4.12)	
	3-4 days/week	1.67 (1.10, 2.54)	1.64 (1.07, 2.52)	1.33 (0.66, 2.69)	1.36 (0.66, 2.82)	2.15 (0.96, 4.79)	2.16 (0.97, 4.80)	
	5 days/week	1.86 (1.21, 2.86)	1.85 (1.20, 2.86)	1.68 (0.86, 3.25)	1.67 (0.84, 3.32)	2.18 (1.04, 4.60)	2.20 (1.04, 4.62)	
P for trend		0.002	0.002	0.008	0.008	0.06	0.06	
Activity score (per unit)		1.19 (1.12, 1.26)	1.19 (1.12, 1.26)	1.20 (1.11, 1.30)	1.19 (1.10, 1.30)	1.18 (1.09, 1.28)	1.18 (1.09, 1.28)	0.79

For peer review only

For peer review only - http://bmjopen.bmj.com/site/about/guidelines.xhtml

* MVPA Moderate-to-vigorous physical activity; Model 1 is adjusted for age and gender; Model 2 is additionally adjusted for BMI and IMD score

For peer review only

Figure S1 Scatter plot of sedentary time by activity score in the observed data (N=1003)

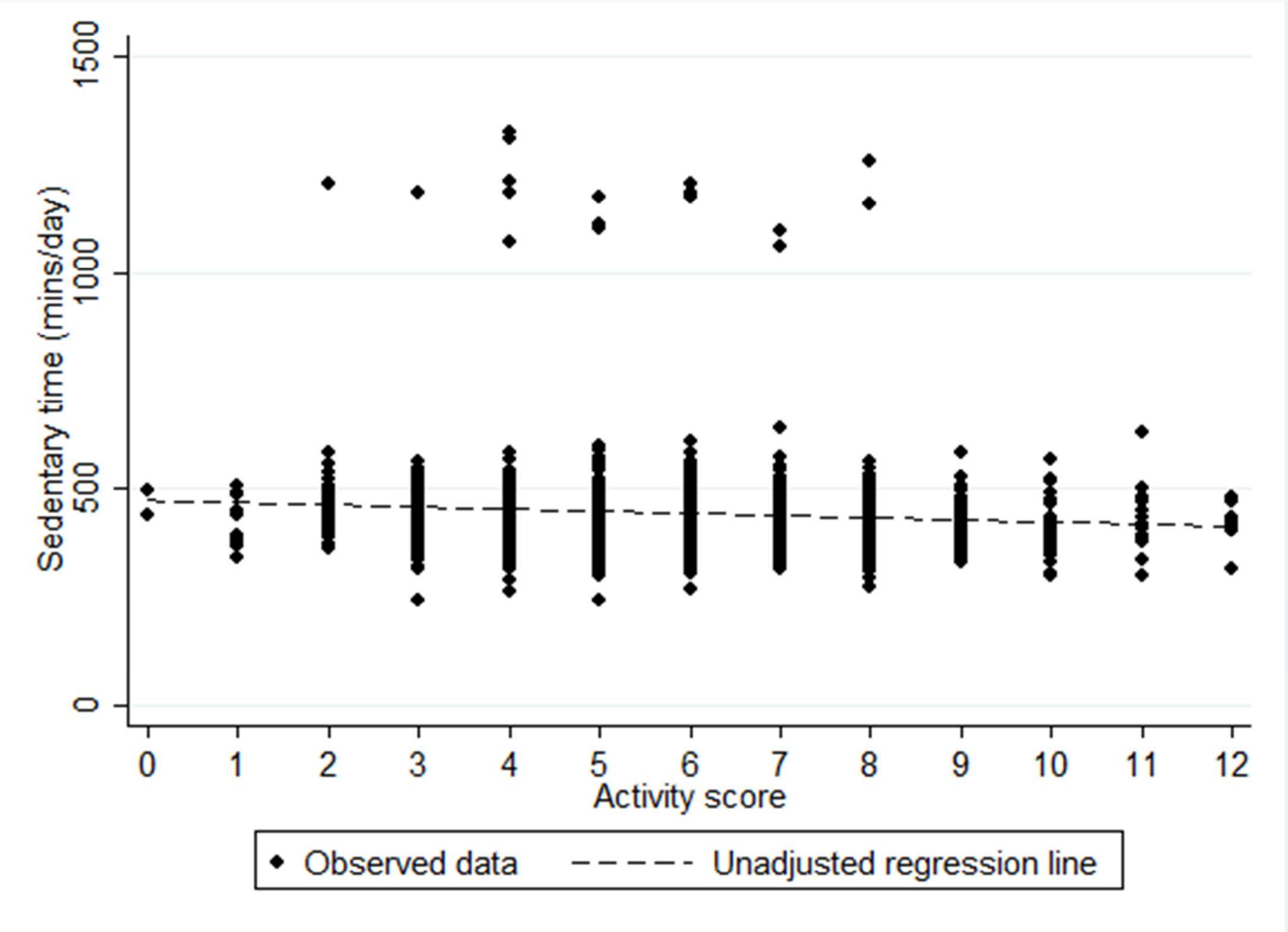

Figure S2 Scatter plot of time spent in moderate-to-vigorous physical activity by activity score in the observed data (N=1003)

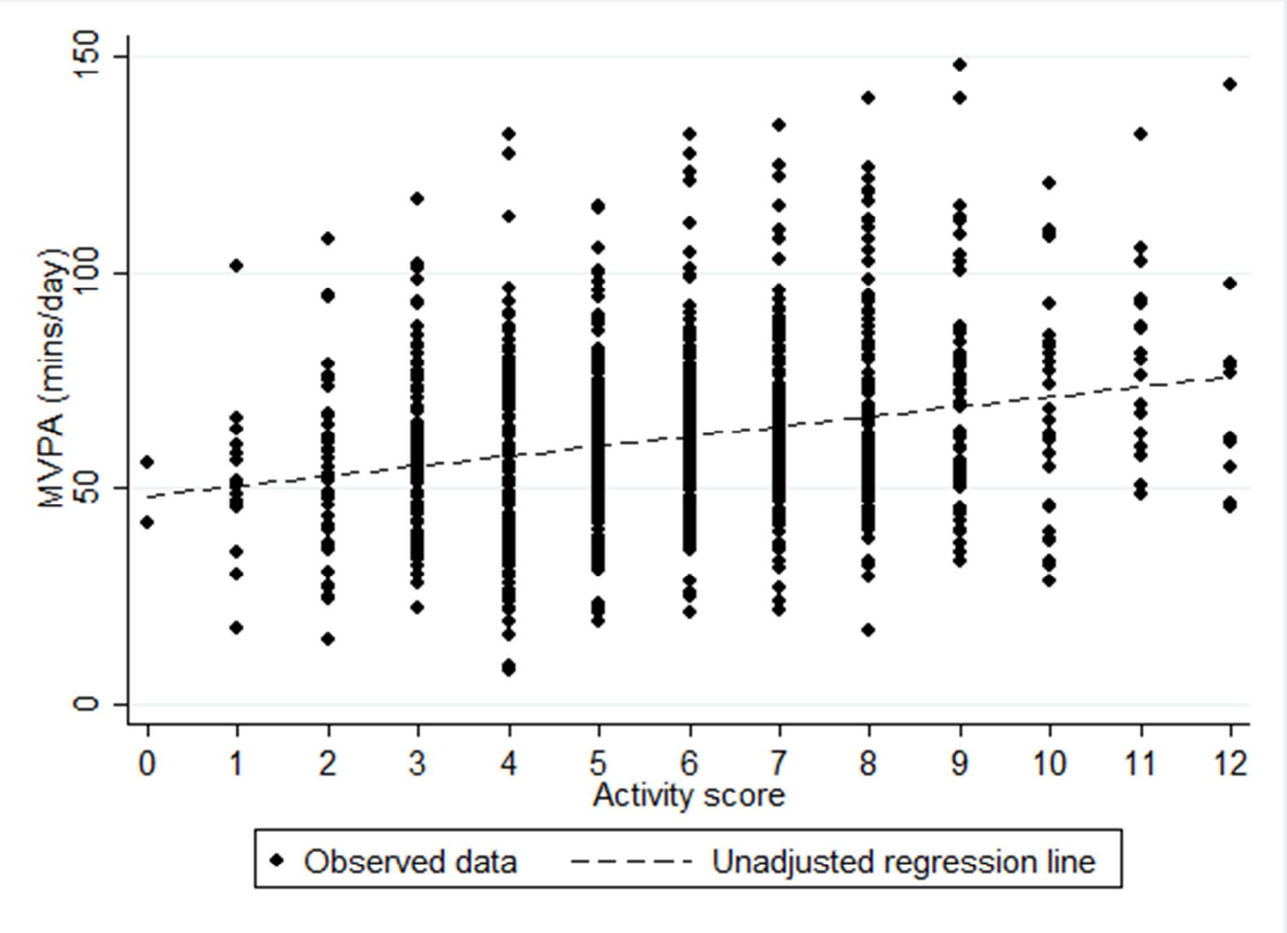

Organised physical activity, neighbourhood play and child physical activity

STROBE Statement—checklist of items that should be included in reports of observational studies

	Item No	Recommendation
**Title and abstract**	1	(*a*) Indicate the study's design with a commonly used term in the title or the abstract Title Page
		(*b*) Provide in the abstract an informative and balanced summary of what was done and what was found – Pages 2-3
**Introduction**		
Background/rationale	2	Explain the scientific background and rationale for the investigation being reported – Pages 5-6
Objectives	3	State specific objectives, including any prespecified hypotheses – Page 6.
**Methods**		
Study design	4	Present key elements of study design early in the paper Pages 6-7.
Setting	5	Describe the setting, locations, and relevant dates, including periods of recruitment, exposure, follow-up, and data collection Page 6.
Participants	6	(*a*) *Cohort study*—Give the eligibility criteria, and the sources and methods of selection of participants. Describe methods of follow-up<br>*Case-control study*—Give the eligibility criteria, and the sources and methods of case ascertainment and control selection. Give the rationale for the choice of cases and controls<br>*Cross-sectional study*—Give the eligibility criteria, and the sources and methods of selection of participants Page 7
		(*b*) *Cohort study*—For matched studies, give matching criteria and number of exposed and unexposed<br>*Case-control study*—For matched studies, give matching criteria and the number of controls per case
Variables	7	Clearly define all outcomes, exposures, predictors, potential confounders, and effect modifiers. Give diagnostic criteria, if applicable - Page 7-8
Data sources/ measurement	8*	For each variable of interest, give sources of data and details of methods of assessment (measurement). Describe comparability of assessment methods if there is more than one group Page 7-8
Bias	9	Describe any efforts to address potential sources of bias – Page 8
Study size	10	Explain how the study size was arrived at - Page 8
Quantitative variables	11	Explain how quantitative variables were handled in the analyses. If applicable, describe which groupings were chosen and why - Pages 8-9
Statistical methods	12	(*a*) Describe all statistical methods, including those used to control for confounding – Pages 8-9
		(*b*) Describe any methods used to examine subgroups and interactions – Pages 8-9
		(*c*) Explain how missing data were addressed - Pages 8-9
		(*d*) *Cohort study*—If applicable, explain how loss to follow-up was addressed<br>*Case-control study*—If applicable, explain how matching of cases and controls was addressed<br>*Cross-sectional study*—If applicable, describe analytical methods taking account of

For peer review only - http://bmjopen.bmj.com/site/about/guidelines.xhtml

		sampling strategy - Page 9
		(e) Describe any sensitivity analyses – Page 10

Continued on next page

For peer review only

For peer review only - http://bmjopen.bmj.com/site/about/guidelines.xhtml

**Results**		
Participants	13*	(a) Report numbers of individuals at each stage of study—eg numbers potentially eligible, examined for eligibility, confirmed eligible, included in the study, completing follow-up, and analysed Table 1
		(b) Give reasons for non-participation at each stage
		(c) Consider use of a flow diagram - NA
Descriptive data	14*	(a) Give characteristics of study participants (eg demographic, clinical, social) and information on exposures and potential confounders – Table 1
		(b) Indicate number of participants with missing data for each variable of interest - Table 1
		(c) *Cohort study*—Summarise follow-up time (eg, average and total amount)
Outcome data	15*	*Cohort study*—Report numbers of outcome events or summary measures over time
		Case-control study—Report numbers in each exposure category, or summary measures of exposure
		Cross-sectional study—Report numbers of outcome events or summary measures Table 1
Main results	16	(*a*) Give unadjusted estimates and, if applicable, confounder-adjusted estimates and their precision (eg, 95% confidence interval). Make clear which confounders were adjusted for and why they were included – Tables 2-4
		(*b*) Report category boundaries when continuous variables were categorized - Tables 2-4
		(*c*) If relevant, consider translating estimates of relative risk into absolute risk for a meaningful time period Discussion – Page 18
Other analyses	17	Report other analyses done—eg analyses of subgroups and interactions, and sensitivity analyses – Supplementary tables
**Discussion**		
Key results	18	Summarise key results with reference to study objectives – Page 21
Limitations	19	Discuss limitations of the study, taking into account sources of potential bias or imprecision. Discuss both direction and magnitude of any potential bias - Page 23
Interpretation	20	Give a cautious overall interpretation of results considering objectives, limitations, multiplicity of analyses, results from similar studies, and other relevant evidence – Page 22
Generalisability	21	Discuss the generalisability (external validity) of the study results Page 22
**Other information**		
Funding	22	Give the source of funding and the role of the funders for the present study and, if applicable, for the original study on which the present article is based – Page 24

*Give information separately for cases and controls in case-control studies and, if applicable, for exposed and unexposed groups in cohort and cross-sectional studies.

Note: An Explanation and Elaboration article discusses each checklist item and gives methodological background and published examples of transparent reporting. The STROBE checklist is best used in conjunction with this article (freely available on the Web sites of PLoS Medicine at http://www.plosmedicine.org/, Annals of Internal Medicine at http://www.annals.org/, and Epidemiology at http://www.epidem.com/). Information on the STROBE Initiative is available at www.strobe-statement.org.

For peer review only

For peer review only - http://bmjopen.bmj.com/site/about/guidelines.xhtml

Associations between participation in organised physical activity in the school or community outside school hours, and neighbourhood play with child physical activity and sedentary time: a cross-sectional analysis of primary school-aged children from the UK

Journal:	*BMJ Open*
Manuscript ID	bmjopen-2017-017588.R1
Article Type:	Research
Date Submitted by the Author:	26-Jun-2017

Complete List of Authors:	Jago, Russell; Univ Bristol, Centre for Exercise, Nutrition & Health Sciences, School for Policy Studies<br>Macdonald-Wallis, Corrie; University of Bristol, Centre for Exercise, Nutrition & Health Sciences, School for Policy Studies, University of Bristol<br>Solomon-Moore, Emma; Univ Bristol, Centre for Exercise, Nutrition & Health Sciences, School for Policy Studies<br>Thompson, Janice; The University of Birmingham, School of Sport, Exercise and Rehabilitation Sciences<br>Lawlor, Debbie; University of Bristol School of Social and Community Medicine, MRC Integrative Epidemiology Unit at the University of Bristol<br>Sebire, Simon; University of Bristol, Centre for Exercise, Nutrition & Health Sciences, School for Policy Studies

**Primary Subject Heading**:	Public health
Secondary Subject Heading:	Paediatrics
Keywords:	Physical activity, children, accelerometer, extra-curricular, play

SCHOLARONE™ Manuscripts

Associations between participation in organised physical activity in the school or community outside school hours, and neighbourhood play with child physical activity and sedentary time: a cross-sectional analysis of primary school-aged children from the UK

Russell Jago [1$], Corrie Macdonald-Wallis [1], Emma Solomon-Moore [1], Janice L. Thompson [2], Deborah A. Lawlor [3, 4] and Simon J. Sebire [1]

[1] Centre for Exercise, Nutrition & Health Sciences, School for Policy Studies, University of Bristol, 8 Priory Road, Bristol, BS8 1TZ, UK.

[2] School of Sport, Exercise and Rehabilitation Sciences, University of Birmingham, Birmingham, B15 2TT, UK.

[3] MRC Integrative Epidemiology Unit at the University of Bristol, Oakfield House, Oakfield Grove, Bristol, BS8 2BN, UK.

[4] School of Social and Community Medicine, University of Bristol, Canynge Hall, Whiteladies Road, Bristol, BS8 2PS, UK.

[$] **Corresponding author:** Prof Russell Jago, Centre for Exercise, Nutrition & Health Sciences, School for Policy Studies, University of Bristol, 8 Priory Road, Bristol, BS8 1TZ, UK. Email: russ.jago@bris.ac.uk; Telephone: +44 (0)117 9546603

Email addresses

RJ:	russ.jago@bris.ac.uk
CMW:	C.Macdonald-Wallis@bristol.ac.uk
ESM:	Emma.solomon@bristol.ac.uk
JLT:	j.thompson.1@bham.ac.uk
DAL:	d.a.lawlor@bristol.ac.uk
SJS:	simon.sebire@bristol.ac.uk

Word count: **Manuscript** (excluding tables) = 3270 words **Abstract** = 298 words

For peer review only - http://bmjopen.bmj.com/site/about/guidelines.xhtml

ABSTRACT

Objectives: Assess the extent to which participation in organised physical activity in the school or community outside school hours, and neighbourhood play, were associated with children's physical activity and sedentary time.

Design: Cross-sectional study.

Setting: Children were recruited from 47 state funded primary schools in Southwest England.

Participants: 1223 8-9 year old children.

Outcome measures: Accelerometer-assessed moderate-to-vigorous-intensity physical activity (MVPA) and sedentary time.

Methods: Children wore an accelerometer and mean minutes of MVPA and sedentary time per day were derived. Children reported their attendance at organised physical activity in the school or community outside school hours, and neighbourhood play, using a piloted questionnaire. Cross-sectional linear and logistic regression were used to examine if attendance frequency at each setting (and all settings combined) was associated with moderate-to-vigorous-intensity physical activity (MVPA) and sedentary time. Multiple imputation methods were used to account for missing data and increase sample size.

Results: Children who attended clubs at school 3-4 days per week obtained an average of 7.58 (95% CI: 2.7 to 12.4) more minutes of MVPA per day than children who never attended. Report of participation in the three other non-school-based activities were similarly associated with MVPA. Evidence for associations with sedentary time was generally weaker. Associations were similar in girls and boys. When the four different contexts were combined, each additional 1-2 activities participated in per week increased participants' odds (OR: 1.18,

For peer review only - http://bmjopen.bmj.com/site/about/guidelines.xhtml

95% CI: 1.12 to 1.26) of meeting the Government recommendations for 60 minutes of MVPA per day.

Conclusion: Participating in organised physical activity at school and in the community is associated with greater physical activity and reduced sedentary time among both boys and girls. All four types of activity contribute to overall physical activity, which provides parents with a range of settings in which to help their child be active.

Key words: Physical activity, children, accelerometer, clubs, extra-curricular, play

For peer review only

For peer review only - http://bmjopen.bmj.com/site/about/guidelines.xhtml

ARTICLE SUMMARY

Strengths and limitations of this study

Strengths

- Accelerometer data from a large sample of Year 4 children.
- Detailed information on organised physical activity in the school or community outside school hours, and neighbourhood play.
- Multiple imputation models to provide estimates for participants with missing data.

Limitations

- Cross-sectional study design.
- Data are from a single UK region.

For peer review only

For peer review only - http://bmjopen.bmj.com/site/about/guidelines.xhtml

INTRODUCTION

Physical activity is associated with improved mental well-being, reduced risk of obesity and lower blood pressure among children.[1] Sedentary time may also be a risk factor for non-communicable diseases but it is not clear if this effect is independent of physical activity.[2-4] The UK Chief Medical Officers recommend that all children and young people should engage in at least an hour per day of moderate-to-vigorous-intensity physical activity (MVPA) and limit sedentary time,[5] however considerable proportions of children do not meet these guidelines.[6] For example, data from the nationally-representative Millennium cohort showed that only 51% of 7-8 year olds met the recommendation.[7] The amount of time children spend engaged in MVPA gradually declines with age, while sedentary time increases.[6 8-11] Strategies to increase children's physical activity are needed.

The majority of interventions to increase children's physical activity have been delivered during school time.[12 13] These interventions have included strategies such as changes to the physical education provision and new educational programmes based on information sharing and personal goal setting.[12-14] Overall, these programmes have tended to report no effect, weak effects or moderate effects in sub-groups.[12-16] Potential reasons for this are the difficulty faced in adding interventions to already full school-curricula and the lack of skills and training that teachers have for delivering a range of activities to engage the majority of children.[17] As such, there is a need to understand the potential of organised physical activity outside school hours to increase MVPA.

After-school programmes have the potential to facilitate physical activity for children, as schools have space in which children can be active, staff who can be trained, and many parents welcome programmes that provide childcare.[16 18-21] Although a number of studies

For peer review only

have examined the potential of delivering such sessions, it is not clear whether attendance at the programmes currently provided by schools is associated with higher overall levels of MVPA.[18-21] There is also a lack of information about how attendance at community-based physical activity clubs contributes to overall MVPA. Furthermore, as not all children attend after-school programmes, it is not clear how other activities such as playing in the neighbourhood or at home in the garden contribute to overall MVPA. A key question, therefore, is whether the frequency of participation in organised physical activity in the school or community after school hours, neighbourhood play or home play is associated with the MVPA and sedentary time of children. Since some children will be active in all four settings, it would also be informative to examine collective participation across all settings.

The aim of this study was to assess among children (8-9 years of age) the extent to which participation in organised physical activity in the school or community outside school hours, and playing with friends or family near the home or in the garden were associated with MVPA and sedentary time. A secondary aim was to examine if there was a cumulative association between participation in the four different types of activities with both MVPA and sedentary time.

METHODS

The current analyses used data from the B-Proact1v study, which has been described in detail elsewhere.[11 22 23] Briefly, the study aimed to examine physical activity behaviours of children and their parents over the course of primary school. Between 2012 and 2013, data were collected from 1299 Year 1 children (5-6 years of age) from 57 schools in Bristol (UK). Between March 2015 and July 2016, all 57 schools were approached to re-join the study when the children were in Year 4 (8-9 years of age), with 47 schools agreeing to take part

For peer review only

(1223 children). The current analyses used data from the Year 4 assessments. The study received ethical approval from the School for Policy Studies Ethics Committee at the University of Bristol, and written parent consent was received for all participants.

Data collection

Data were collected at schools, with children asked to complete a brief questionnaire. As indicators of organised physical activity outside school hours in school and in the community respectively, we asked "How often do you attend…a) a sport or exercise club at school (NOT including PE)? and b) a sport or exercise club at places other than your school (like a football club or ballet)?" To indicate neighbourhood play outside and within the home we asked "How much do you play with your friends and family… a) outside near your home? and b) in your home or garden?". These questions each had four response options: "Never", "1-2 days per week", "3-4 days per week" or "5 days per week". We assigned these 0, 1, 2 and 3 points respectively and summed responses to derive an overall activity score ranging from 0 to 12, with a higher value indicating a higher frequency of activity participation.

Child height was measured to the nearest 0.1cm using a SECA Leicester stadiometer (HAB International, Northampton). Weight was recorded to the nearest 0.1kg using a SECA 899 digital scale (HAB International, Northampton). Child Body Mass Index (BMI = kg/m^2) was then calculated and converted to an age- and gender-specific standard deviation score.[24 25] Children wore a waist-worn ActiGraph wGT3X-BT accelerometer for five days including two weekend days. Parents provided demographic information via a questionnaire, including child gender and date of birth. Where children's date of birth was missing (20.5% of children), they were assigned the median age of 9.0 years. Indices of Multiple Deprivation (IMD) scores, based upon the English Indices of Deprivation

For peer review only - http://bmjopen.bmj.com/site/about/guidelines.xhtml

(http://data.gov.uk/dataset/index-of-multiple-deprivation), were assigned to each child based on their reported home postcode, where higher IMD scores indicate a greater level of deprivation.

Accelerometer data reduction

Accelerometer data were processed using Kinesoft (v3.3.75; Kinesoft, Saskatchewan, Canada). At least three valid days of data were required for accelerometer data to be considered complete for a given child and included in analysis, where a valid day was defined as at least 500 minutes of data, after excluding intervals of ≥60 minutes of zero counts allowing up to two minutes of interruptions. We recognise that there is considerable variation in the number of minutes of accelerometer data that are required to be considered representative of a valid day.[26] These have ranged from 360 minutes per day which has been used for 6 to 8 year old children,[27] to 800 minutes which has been used for older children.[28 29] Within the field there is no consensus on the minimum number of minutes per day that are needed for a day to be considered valid. We, therefore, adopted a 500 minute per day threshold to ensure that our data are comparable to the methods employed by the International Children's Accelerometer Database,[6] which has pooled data from over 27,000 children across 20 large global cohorts. The child's average number of sedentary and MVPA minutes per day were derived using population-specific cut points for children.[30] We also derived a binary variable indicating whether the child's average daily MVPA was greater than the 60 minutes per day recommended by the UK government.[5]

Analysis

For peer review only - http://bmjopen.bmj.com/site/about/guidelines.xhtml

The associations of child characteristics (gender, age, BMI z-score, and IMD) with activity participation were examined in the observed data using t-tests, Pearson's correlation coefficients, chi-squared tests and one-way analysis of variance as appropriate.

Multiple imputation of missing data was used to create 20 imputed datasets for the 1223 Year 4 children. We used 20 cycles of regression switching and combined regression coefficients across datasets using Rubin's rules.[31] We imputed separately for boys and girls to allow for associations to differ by gender. All exposures (organised physical activity attendance and neighbourhood play), outcomes (sedentary time and MVPA), potential confounders (gender, age, BMI z-score, and IMD) and child's school were included in multiple imputation models, and achievement of the MVPA guideline and overall activity score were imputed passively. Any children with less than three valid days of accelerometer data had their accelerometer measures imputed.

We examined the pairwise associations of the activity participation variables by dichotomising, cross-tabulating and fitting unadjusted logistic regression models of one frequency variable on another.

We used linear regression models to examine the associations of activity participation and the overall activity score with the child's average sedentary and MVPA minutes per day, and logistic regression models to examine associations with achievement of the MVPA guideline. In Model 1 we adjusted for gender and age, and in Model 2 we adjusted additionally for BMI z-score and IMD. To account for the clustering of children in schools and the associated potential to underestimate the standard errors which are used to compute the 95% confidence

For peer review only - http://bmjopen.bmj.com/site/about/guidelines.xhtml

intervals and p-values, robust standard errors, which took account of the school level clustering, were used for all models. Combined Wald tests were used to test for evidence of interaction between the child's gender and the exposure of interest.

We predicted the children's mean number of sedentary and MVPA minutes per day by frequency of participation in each activity based on linear combinations of the regression coefficients from fully-adjusted models (Model 2).

Regression analyses were repeated restricting to the children who had complete data for all exposures, outcomes and co-variables and compared with the multiple imputation analysis. We also produced scatter plots of sedentary time and MVPA by the overall activity score in the observed data.

A sensitivity analysis was performed including accelerometer data for any children who had at least one valid day of measures to assess whether only including accelerometer data for children who recorded at least three valid days influenced our results. All analyses were performed in Stata version 14.0 (StataCorp, 2015).

RESULTS

The distributions of characteristics of the children in the observed data, multiple imputation datasets and subset with complete information are shown in Table 1. All characteristics showed similar distributions in each of the datasets and had only a small proportion of missing data (maximum 16.1% for accelerometer measures).

For peer review only - http://bmjopen.bmj.com/site/about/guidelines.xhtml

Table 1 Characteristics of children who took part in the Year 4 phase of the B-Proact1v Study (observed and multiple imputation) N=1223)

Child Characteristic		Observed data		Imputed data (N=1223)	Complete data (N=987)
		N available	Mean (SD) or %	Mean (SD) or %	Mean (SD) or %
Sedentary time at Year 4 (mins/day)		1026	445.4 (115.4)	444.7 (120.1)	446.0 (116.9)
MVPA at Year 4 (mins/day)		1026	61.6 (21.9)	61.9 (22.5)	61.8 (21.8)
Met MVPA guidelines at Year 4	No	1163	53.2	52.6	53.4
	Yes		46.8	47.4	46.6
Gender	Boy	1223	45.5	45.5	44.5
	Girl		54.5	54.5	55.5
Age at Year 4 (years)		1223	9.03 (0.41)	9.03 (0.41)	9.03 (0.43)
BMI z score at Year 4		1208	0.35 (1.08)	0.36 (1.08)	0.31 (1.07)
IMD score at Year 4		1204	15.9 (14.1)	15.9 (14.2)	15.3 (13.6)
Frequency child attends sport/exercise club at school	Never	1215	27.8	27.9	28.2
	1-2 days per week		45.5	45.5	45.8
	3-4 days per week		16.1	16.1	16.6
	5 days per week		10.5	10.5	9.4
Frequency child attends sport/exercise club outside school	Never	1214	20.6	20.6	19.4
	1-2 days per week		50.2	50.2	51.0
	3-4 days per week		20.8	20.9	21.5
	5 days per week		8.3	8.3	8.2
Frequency child plays with friends/family outside near home	Never	1205	6.3	6.4	6.5
	1-2 days per week		33.7	33.7	34.3
	3-4 days per week		29.0	28.9	29.7
	5 days per week		31.0	30.9	29.5
Frequency child plays with friends/family in home or garden	Never	1199	9.6	9.7	8.8
	1-2 days per week		34.5	34.6	35.0
	3-4 days per week		26.8	26.7	27.8
	5 days per week		29.1	29.0	28.5
Activity frequency score		1193	5.88 (2.29)	5.86 (2.29)	5.84 (2.26)

For peer review only

For peer review only - http://bmjopen.bmj.com/site/about/guidelines.xhtml

Associations of child characteristics with activity participation

Table S1 shows child activity attendance by gender. Girls tended to report lower frequencies of participating in organised physical activity in the school or community outside school hours, and had a lower mean overall activity score. There was no gender difference in friends/family play either in or outside of the home. The associations of other child characteristics with activity participation are shown in Table S2. There was some evidence that children who more frequently attended a sport/exercise club outside of school had a higher mean age. Child BMI was not strongly associated with any particular activity, but there was weak evidence that the overall activity score decreased with increasing BMI Z-score. Children who reported attendance of “Never” or “5 days/week” generally had the highest IMD scores, suggesting a U-shaped association, and there was a negative correlation between the overall activity score and IMD.

Inter-relationships of activity participation frequencies

Participating in one type of activity more frequently was generally associated with a higher frequency of participation in each of the others, except that attendance at a sport/exercise club outside of school was not associated with playing outside near the home (Table S3).

Associations of activity participation with sedentary time and MVPA

There was a negative correlation of the overall activity score with sedentary time and a positive correlation between the overall activity score and MVPA (Figures S1 and S2).

Table 2 shows the mean difference in sedentary time by activity participation and overall activity score in the multiple imputation datasets, and Figure 1 shows the predicted sedentary time by activity participation. Sedentary time decreased on average with increasing frequency

For peer review only - http://bmjopen.bmj.com/site/about/guidelines.xhtml

of attending sport/exercise clubs either at school or outside of school and with increasing frequency of playing with friends/family outside near the home in regression models adjusted for gender and age (Model 1). The association between sport/exercise club attendance outside of school and sedentary time weakened slightly on additional adjustment for BMI Z-score and IMD (Model 2) but other associations remained. An increase in children's overall activity score was also strongly associated with a reduction in sedentary time in both models. However, there was no evidence of an association between playing with friends/family at home and sedentary time. Associations did not differ between boys and girls. Findings were similar when restricted to children who had complete data (Table S4).

For peer review only

For peer review only - http://bmjopen.bmj.com/site/about/guidelines.xhtml

Table 2 Mean difference in the children's average sedentary minutes per day associated with different activities using multiple imputation (N=1223)*

Exposure		Sedentary time (minutes/day): mean difference (95% confidence interval)						P for gender interaction
		All (N=1223)		Boys (N=556)		Girls (N=667)		
		Model 1	Model 2	Model 1	Model 2	Model 1	Model 2	
Frequency child attends sport/exercise club at school	Never (ref)	0	0	0	0	0	0	0.69
	1-2 days/week	-4.2 (-22.5, 14.0)	-3.0 (-21.4, 15.4)	7.9 (-16.6, 32.3)	8.9 (-15.9, 33.7)	-13.0 (-38.3, 12.4)	-11.5 (-37.5, 14.4)	
	3-4 days/week	-31.1 (-50.7, -11.5)	-28.8 (-46.4, -11.3)	-18.7 (-41.8, 4.4)	-16.9 (-41.2, 7.3)	-40.1 (-71.7, -8.5)	-37.3 (-65.4, -9.2)	
	5 days/week	-18.7 (-42.9, 5.6)	-18.8 (-42.1, 4.5)	-9.6 (-37.7, 18.4)	-7.3 (-34.9, 20.4)	-26.0 (-66.8, 14.8)	-33.3 (-82.0, 15.5)	
P for trend		0.02	0.01	0.17	0.23	0.05	0.04	
Frequency child attends sport/exercise club outside of school	Never (ref)	0	0	0	0	0	0	0.81
	1-2 days/week	14.8 (-9.1, 38.7)	18.0 (-8.9, 45.0)	21.5 (-7.4, 50.5)	25.9 (-4.5, 56.4)	11.0 (-19.9, 42.0)	13.6 (-20.5, 47.7)	
	3-4 days/week	-7.2 (-27.0, 12.6)	-1.5 (-23.4, 20.4)	-1.0 (-32.8, 30.8)	4.8 (-27.1, 36.6)	-10.5 (-30.5, 9.5)	-4.1 (-27.3, 19.0)	
	5 days/week	-19.9 (-41.1, 1.2)	-15.5 (-37.7, 6.8)	-12.6 (-42.4, 17.1)	-7.7 (-36.5, 21.1)	-25.2 (-55.0, 4.6)	-20.6 (-52.1, 11.0)	
P for trend		0.02	0.07	0.12	0.16	0.07	0.23	
Frequency child plays with friends/family outside near home	Never (ref)	0	0	0	0	0	0	0.56
	1-2 days/week	0.77 (-28.5, 30.0)	2.3 (-28.0, 32.7)	19.2 (-16.5, 55.0)	21.2 (-14.4, 56.8)	-16.6 (-63.1, 30.0)	-15.6 (-65.0, 33.9)	
	3-4 days/week	-5.2 (-29.9, 19.6)	-2.8 (-29.4, 23.7)	7.6 (-28.4, 43.5)	10.6 (-25.5, 46.6)	-17.6 (-65.8, 30.5)	-16.1 (-67.4, 35.3)	
	5 days/week	-23.4 (-49.0, 2.1)	-24.1 (-50.8, 2.7)	-11.0 (-45.2, 23.2)	-10.2 (-44.4, 24.0)	-35.4 (-79.9, 9.0)	-37.8 (-86.6, 10.9)	
P for trend		0.004	0.001	0.07	0.05	0.02	0.02	
Frequency child plays with friends/family in home/garden	Never (ref)	0	0	0	0	0	0	0.90
	1-2 days/week	16.2 (-13.8, 46.3)	17.9 (-12.5, 48.2)	19.2 (-22.9, 61.3)	20.6 (-21.5, 62.7)	13.1 (-25.8, 52.0)	14.9 (-24.3, 54.1)	
	3-4 days/week	-3.9 (-28.8, 20.9)	-0.7 (-26.7, 25.4)	3.9 (-30.5, 38.4)	6.5 (-28.7, 41.7)	-10.7 (-46.6, 25.3)	-6.4 (-42.9, 30.1)	
	5 days/week	5.3 (-22.9, 33.5)	6.1 (-22.5, 34.7)	9.9 (-32.5, 52.3)	11.6 (-31.2, 54.4)	1.2 (-39.2, 41.5)	0.6 (-40.2, 41.5)	
P for trend		0.49	0.49	0.85	0.91	0.49	0.45	
Activity score (per unit)		-4.8 (-7.3, -2.3)	-4.6 (-7.0, -2.1)	-3.8 (-7.7, 0.1)	-3.4 (-7.2, 0.4)	-5.9 (-10.0, -1.7)	-5.9 (-10.2, -1.6)	0.41

* Model 1 is adjusted for age and gender; Model 2 is additionally adjusted for BMI and IMD score

For peer review only

For peer review only - http://bmjopen.bmj.com/site/about/guidelines.xhtml

The mean difference in MVPA by each of the activity variables in the multiple imputation data is shown in Table 3, with predicted MVPA by activity participation presented in Figure 2. A higher frequency of attending sport/exercise clubs either at school or outside of school and of play either outside or in the home/garden were all associated with greater MVPA on average in Models 1 and 2. Associations were similar in boys and girls. A higher overall activity score was also associated with greater MVPA, with some evidence that this association was stronger in boys than in girls. Associations were similar when restricted to children with complete data (Table S5).

For peer review only

For peer review only - http://bmjopen.bmj.com/site/about/guidelines.xhtml

Table 3 Mean difference in the children's average MVPA minutes per day associated with different activities using multiple imputation (N=1223)*

Exposure		Moderate-to-vigorous physical activity (minutes/day): mean difference (95% confidence interval)						
		All (N=1223)		Boys (N=556)		Girls (N=667)		P for gender interaction
		Model 1	Model 2	Model 1	Model 2	Model 1	Model 2	
Frequency child attends sport/exercise club at school	Never (ref)	0	0	0	0	0	0	0.64
	1-2 days/week	3.7 (0.3, 7.2)	3.6 (0.1, 7.0)	4.4 (-2.1, 10.9)	4.1 (-2.3, 10.5)	3.2 (-0.3, 6.7)	3.1 (-0.3, 6.6)	
	3-4 days/week	7.8 (2.9, 12.6)	7.5 (2.7, 12.4)	10.1 (2.6, 17.7)	9.6 (2.0, 17.2)	5.9 (0.9, 10.8)	5.8 (0.9, 10.7)	
	5 days/week	5.9 (-0.1, 11.8)	5.7 (-0.3, 11.7)	7.5 (-0.4, 15.4)	7.0 (-1.1, 15.0)	3.8 (-3.7, 11.3)	3.6 (-4.1, 11.4)	
P for trend		0.007	0.01	0.02	0.03	0.05	0.06	
Frequency child attends sport/exercise club outside of school	Never (ref)	0	0	0	0	0	0	0.80
	1-2 days/week	0.9 (-2.5, 4.3)	0.7 (-2.9, 4.4)	1.8 (-4.7, 8.3)	1.3 (-5.5, 8.2)	0.5 (-3.3, 4.4)	0.5 (-3.4, 4.5)	
	3-4 days/week	6.9 (2.4, 11.5)	6.5 (1.9, 11.2)	8.9 (1.0, 16.8)	8.1 (0.1, 16.0)	5.5 (0.9, 10.1)	5.6 (1.1, 10.2)	
	5 days/week	9.9 (3.8, 16.0)	9.6 (3.3, 15.9)	11.8 (3.4, 20.1)	11.2 (2.4, 19.9)	8.2 (0.7, 15.7)	8.2 (0.8, 15.7)	
P for trend		<0.001	<0.001	0.001	0.002	0.007	0.006	
Frequency child plays with friends/family outside near home	Never (ref)	0	0	0	0	0	0	0.19
	1-2 days/week	0.7 (-4.6, 6.0)	0.4 (-4.9, 5.6)	0.0 (-9.3, 9.2)	-0.5 (-9.9, 8.9)	1.4 (-6.0, 8.8)	1.3 (-6.2, 8.7)	
	3-4 days/week	5.1 (-0.9, 11.1)	4.8 (-1.1, 10.7)	6.1 (-4.1, 16.3)	5.7 (-4.5, 15.9)	4.4 (-3.5, 12.3)	4.3 (-3.7, 12.2)	
	5 days/week	9.6 (4.0, 15.3)	9.5 (3.9, 15.2)	13.0 (3.3, 22.8)	12.9 (3.0, 22.7)	6.7 (-0.7, 14.0)	6.6 (-0.9, 14.0)	
P for trend		<0.001	<0.001	<0.001	<0.001	0.005	0.006	
Frequency child plays with friends/family in home/garden	Never (ref)	0	0	0	0	0	0	0.38
	1-2 days/week	2.0 (-2.4, 6.4)	1.6 (-2.7, 6.0)	-0.1 (-8.4, 8.3)	-0.6 (-8.9, 7.7)	3.8 (-2.2, 9.8)	3.6 (-2.4, 9.7)	
	3-4 days/week	5.6 (0.9, 10.3)	5.3 (0.4, 10.2)	5.6 (-3.1, 14.3)	5.4 (-3.5, 14.4)	5.7 (-0.6, 11.9)	5.5 (-0.9, 12.0)	
	5 days/week	7.4 (3.0, 11.8)	7.1 (2.7, 11.6)	8.3 (0.1, 16.6)	7.7 (-0.7, 16.1)	6.6 (0.7, 12.5)	6.5 (0.6, 12.5)	

For peer review only

For peer review only - http://bmjopen.bmj.com/site/about/guidelines.xhtml

P for trend	<0.001	<0.001	0.004	0.006	0.02	0.02	
Activity score (per unit)	2.1 (1.4, 2.7)	2.0 (1.4, 2.7)	2.6 (1.7, 3.4)	2.5 (1.6, 3.4)	1.6 (0.8, 2.3)	1.6 (0.8, 2.3)	0.06

*MVPA Moderate-to-vigorous physical activity; Model 1 is adjusted for age and gender; Model 2 is additionally adjusted for BMI and IMD score

For peer review only

The associations of activity variables with achievement of the hour per day government guideline in the multiple imputation data are shown in Table 4. A higher frequency of participation in any of the activities, or an increase in the overall activity score, was associated with increased odds of meeting the government guideline in both models. Associations were similar in girls and boys. A unit increase in the activity score was associated with around an 18% increase in the odds of achieving 60 minutes of MVPA per day. Findings were unchanged when restricting to those with complete data (Table S6).

For peer review only

For peer review only - http://bmjopen.bmj.com/site/about/guidelines.xhtml

Table 4 Odds ratios for achieving 60 minutes of MVPA per day associated with different activities using multiple imputation (N=1223)*

Exposure		Meeting government guideline: odds ratio (95% confidence interval)						P for gender interaction
		All (N=1223)		Boys (N=556)		Girls (N=667)		
		Model 1	Model 2	Model 1	Model 2	Model 1	Model 2	
Frequency child attends sport/exercise club at school	Never (ref)	1	1	1	1	1	1	0.34
	1-2 days/week	1.33 (0.98, 1.81)	1.31 (0.97, 1.78)	1.10 (0.65, 1.86)	1.07 (0.63, 1.81)	1.54 (1.06, 2.23)	1.54 (1.07, 2.21)	
	3-4 days/week	1.87 (1.16, 3.01)	1.83 (1.14, 2.93)	2.22 (1.09, 4.53)	2.14 (1.04, 4.40)	1.63 (0.94, 2.85)	1.63 (0.94, 2.82)	
	5 days/week	1.69 (1.03, 2.78)	1.67 (1.02, 2.76)	1.61 (0.83, 3.09)	1.55 (0.79, 3.02)	1.76 (0.84, 3.69)	1.77 (0.84, 3.73)	
P for trend		0.01	0.01	0.03	0.05	0.05	0.05	
Frequency child attends sport/exercise club outside of school	Never (ref)	1	1	1	1	1	1	0.93
	1-2 days/week	1.14 (0.80, 1.64)	1.13 (0.77, 1.64)	1.21 (0.74, 1.98)	1.18 (0.70, 1.99)	1.11 (0.68, 1.80)	1.12 (0.69, 1.82)	
	3-4 days/week	1.82 (1.22, 2.72)	1.75 (1.16, 2.65)	2.07 (1.11, 3.87)	1.95 (1.03, 3.67)	1.66 (0.99, 2.78)	1.69 (1.01, 2.83)	
	5 days/week	2.70 (1.57, 4.62)	2.63 (1.51, 4.58)	3.24 (1.49, 7.07)	3.15 (1.41, 7.06)	2.30 (1.12, 4.72)	2.33 (1.14, 4.77)	
P for trend		<0.001	<0.001	<0.001	0.001	0.009	0.008	
Frequency child plays with friends/family outside near home	Never (ref)	1	1	1	1	1	1	0.74
	1-2 days/week	1.15 (0.70, 1.88)	1.12 (0.69, 1.82)	0.98 (0.46, 2.09)	0.94 (0.43, 2.06)	1.47 (0.62, 3.45)	1.47 (0.63, 3.43)	
	3-4 days/week	1.88 (1.12, 3.16)	1.84 (1.11, 3.06)	1.62 (0.73, 3.58)	1.59 (0.70, 3.58)	2.32 (0.91, 5.91)	2.32 (0.92, 5.88)	
	5 days/week	2.11 (1.26, 3.52)	2.10 (1.26, 3.49)	2.08 (0.92, 4.69)	2.07 (0.91, 4.74)	2.35 (0.93, 5.99)	2.36 (0.93, 6.02)	
P for trend		<0.001	<0.001	0.003	0.002	0.01	0.02	
Frequency child plays with friends/family in home/garden	Never (ref)	1	1	1	1	1	1	0.21
	1-2 days/week	1.27 (0.84, 1.91)	1.23 (0.81, 1.86)	0.94 (0.52, 1.72)	0.90 (0.49, 1.64)	1.75 (0.84, 3.64)	1.75 (0.84, 3.65)	
	3-4 days/week	1.51 (1.00, 2.28)	1.46 (0.96, 2.23)	1.29 (0.70, 2.40)	1.28 (0.68, 2.44)	1.80 (0.86, 3.80)	1.80 (0.86, 3.79)	
	5 days/week	1.71 (1.14, 2.56)	1.67 (1.11, 2.53)	1.71 (0.90, 3.25)	1.62 (0.83, 3.14)	1.80 (0.88, 3.69)	1.81 (0.88, 3.70)	

For peer review only - http://bmjopen.bmj.com/site/about/guidelines.xhtml

P for trend	0.005	0.006	0.01	0.02	0.21	0.21	
Activity score (per unit)	1.18 (1.12, 1.25)	1.18 (1.11, 1.25)	1.21 (1.12, 1.30)	1.20 (1.11, 1.30)	1.16 (1.07, 1.25)	1.16 (1.07, 1.25)	0.52

* MVPA Moderate-to-vigorous physical activity; Model 1 is adjusted for age and gender; Model 2 is additionally adjusted for BMI and IMD score

For peer review only

For peer review only - http://bmjopen.bmj.com/site/about/guidelines.xhtml

Sensitivity Analysis

When we changed the inclusion criteria for accelerometer measures from three to one valid day, findings were largely unchanged, except that boys showed a stronger association between sport/exercise club attendance at their school and MVPA than girls (p for interaction = 0.03 in multiple imputation data).

DISCUSSION

The findings demonstrate that increased participation in organised physical activity at school and in the community are associated with greater overall physical activity and reduced sedentary time among both boys and girls. Specifically, a child who attends a school-based club 3-4 days per week obtained 7.8 more minutes of MVPA per day than a child who did not attend at all, with attendance of five days a week at a sport/exercise club outside of school associated with 10.9 more minutes of MVPA than children who never attended clubs. There were comparable patterns for engagement in non-organised activity at home or in the neighbourhood, both were associated with increased MVPA, but only activity outside of the home was associated with reduced sedentary time. When the four different contexts of physical activity were combined, the analyses showed that each additional 1-2 activities participated in per week increased the odds of meeting the Chief Medical Officers' recommendation of 60 minutes of MVPA per day by 18%. Thus, encouraging children to attend after-school and community-based physical activity clubs, as well as to play at home and in the neighbourhood is critical for helping children to increase MVPA. Moreover, in light of the relative consistency in findings for each of the four forms of physical activity, the message to parents should be that physical activity can be accumulated in all four settings which allows them to find a balance that works for their family. For some families with working parents, after-school programmes may be the key activity to focus on, whereas for

For peer review only - http://bmjopen.bmj.com/site/about/guidelines.xhtml

other families encouraging children to play in the neighbourhood is likely to be useful for maximising physical activity. Furthermore, as there was little evidence that play at home was associated with a reduction in sedentary time, it is also important to examine ways of encouraging non-sedentary activities within the home.

The findings in this paper support previous research which has shown that introducing extra-curricular clubs into the school day can promote increased physical activity among primary school-aged children.[32] The study is in broad agreement with the body of work, which has shown that risky outdoor play and higher independent mobility are associated with higher levels of physical activity among children.[33-36] These findings complement these bodies of work by showing an additional effect of accumulating physical activity across different settings to maximise the overall amount of physical activity in which children engage.

The UK Childhood Obesity strategy recommends that all primary schools should provide at least 30 minutes per day of physical activity opportunities across the curriculum, break times and extra-curricular activities.[37] The data presented here show that in the imputed dataset 72.2% of Year 4 children were attending school-based programmes at least once per week and 10.5% were attending five days per week. Previous research has shown that in the UK, after-school clubs for primary school children tend to be dominated by team sports, such as football and rugby, with limited provision for non-competitive physical activities.[38] Thus, increasing the number and variety of sessions that children attend and improving the quality of those sessions is likely to provide a cost effective means of increasing children's physical activity. This hypothesis is consistent with the recent Theory of Expanded, Extended and Enhanced Opportunities (TEO), which suggests that the most effective means of increasing children's physical activity will be provided by extending and expanding current

For peer review only - http://bmjopen.bmj.com/site/about/guidelines.xhtml

For peer review only

provision.[19] Thus, schools and community groups should be encouraged to extend current after-school provision to more children, diversify the activities to interest more pupils (preferably involving pupils in deciding what activities to offer), and enhance the quality of provision to maximise the amount of activity obtained. These relatively simple changes could be made at each school and would provide scalable ways for increasing overall levels of physical activity and contributing to the UK government's goal of reducing the prevalence of childhood obesity.

Strengths and limitations

The major strength of this study is the large sample size and provision of detailed information about participation in four different physical activity settings alongside accelerometer-assessed physical activity. In addition, the use of multiple imputation models to provide estimates for participants with missing data using a robust methodology has enabled us to maximise the sample for analysis. The study is limited by the cross-sectional design, which limits the ability to infer causation between frequency of participation in different settings and levels of physical activity. All questions were self-reported and it is possible that some were recalled more accurately than others. Moreover, as the questions used were developed for this project we do not have information on the reliability and validity of the scale. The report of play within the home is likely to include both sedentary and physically active forms of play, as the question included play indoors, which could be expected to be more sedentary, as well as outdoors in the garden, which is likely to be more active. Equally, as the question focussed on play with friends or family we do not have any information about individual play, and we were unable to disentangle these inter-related issues. We also cannot rule out the possibility of residual confounding, but have adjusted for several key potential confounding

For peer review only - http://bmjopen.bmj.com/site/about/guidelines.xhtml

variables in order to minimise this. The study is also drawn from the greater Bristol area in the UK and as such our ability to extend findings to other settings and countries is limited.

CONCLUSIONS

Participation in organised physical activity at school and in the community is associated with greater physical activity and reduced sedentary time among both boys and girls. In light of the challenges of promoting physical activity during school time, parents should encourage children to attend after school clubs, attend community activity groups and play in the neighbourhood to help their children to meet physical activity guidelines. The data show that all four types of activity contribute similarly to overall physical activity and there is therefore an opportunity for families to find the best mix of options for them.

ACKNOWLEDGEMENTS

This work was supported by grants from the British Heart Foundation (ref PG/11/51/28986 and SP 14/4/31123). DAL works in a unit that receives funding from the University of Bristol and UK Medical Research Council (MC_UU_1201/5); she is also a UK National Institute of Health and Research Senior Investigator (NF-SI-0166-10196). The funders had no involvement in data analysis, data interpretation or writing of the paper.

We would like to thank all of the families and schools that have taken part in the B-PROACT1V project. We would also like to thank all current and previous members of the research team who are not authors on this paper.

COMPETING INTERESTS

All authors have completed the ICMJE uniform disclosure form at www.icmje.org/coi_disclosure.pdf and declare: all authors had financial support from the

For peer review only - http://bmjopen.bmj.com/site/about/guidelines.xhtml

British Heart Foundation for the submitted work; no financial relationships with any organisations that might have an interest in the submitted work in the previous three years; no other relationships or activities that could appear to have influenced the submitted work.

CONTRIBUTORS

Conception / design: RJ, ESM, JLT, DAL and SJS.

Data analysis / acquisition/ interpretation: RJ, CMW, ESM and DAL

Drafting / revising critically for important content: All authors.

Final approval: All authors.

Accountability for study and manuscript: RJ.

DATA SHARING STATEMENT: The datasets generated during the current study are not publicly available as the project is ongoing and data are not ready for archiving. We will consider reasonable requests for access to the data once the project is complete in 2019.

REFERENCES

1. Strong WB, Malina RM, Blimkie CJ, et al. Evidence based physical activity for school-age youth. J Pediatr 2005;**146**(6):732-7

2. Carson V, Hunter S, Kuzik N, et al. Systematic review of sedentary behaviour and health indicators in school-aged children and youth: an update. Applied physiology, nutrition, and metabolism = Physiologie appliquee, nutrition et metabolisme 2016;**41**(6 Suppl 3):S240-65 doi: 10.1139/apnm-2015-0630[published Online First: Epub Date]|.

For peer review only - http://bmjopen.bmj.com/site/about/guidelines.xhtml

3. Ekelund U, Luan J, Sherar LB, et al. Moderate to vigorous physical activity and sedentary time and cardiometabolic risk factors in children and adolescents. JAMA 2012;**307**(7):704-12 doi: 10.1001/jama.2012.156[published Online First: Epub Date]|.

4. Ekelund U, Steene-Johannessen J, Brown WJ, et al. Does physical activity attenuate, or even eliminate, the detrimental association of sitting time with mortality? A harmonised meta-analysis of data from more than 1 million men and women. Lancet 2016;**388**(10051):1302-10 doi: 10.1016/S0140-6736(16)30370-1[published Online First: Epub Date]|.

5. Department of Health PA, Health Improvement and Protection,. Start Active, Stay Active: A report on physical activity from the four home countries' Chief Medical Officers. London, 2011.

6. Cooper AR, Goodman A, Page AS, et al. Objectively measured physical activity and sedentary time in youth: the International children's accelerometry database (ICAD). Int J Behav Nutr Phys Act 2015;**12**:113 doi: 10.1186/s12966-015-0274-5[published Online First: Epub Date]|.

7. Griffiths LJ, Cortina-Borja M, Sera F, et al. How active are our children? Findings from the Millennium Cohort Study. BMJ open 2013;**3**(8):e002893 doi: 10.1136/bmjopen-2013-002893[published Online First: Epub Date]|.

8. Nader PR, Bradley RH, Houts RM, McRitchie SL, O'Brien M. Moderate-to-vigorous physical activity from ages 9 to 15 years. JAMA 2008;**300**(3):295-305

9. Biddle SJ, Pearson N, Ross GM, Braithwaite R. Tracking of sedentary behaviours of young people: A systematic review. Prev Med 2010;**51**:345–51

10. Farooq MA, Parkinson KN, Adamson AJ, et al. Timing of the decline in physical activity in childhood and adolescence: Gateshead Millennium Cohort Study. Br J Sports Med 2017:1-6 doi: doi:10.1136/bjsports-2016-096933[published Online First: Epub Date]|.

For peer review only

11. Jago R, Solomon-Moore E, McDonald-Wallis C, Sebire SJ, Thompson JL, Lawlor DA. Change in children's physical activity and sedentary time between Year 1 and Year 4 of primary school in the B-PROACT1V cohort. Int J Behav Nutr Phys Act In Press

12. Metcalf B, Henley W, Wilkin T. Effectiveness of intervention on physical activity of children: systematic review and meta-analysis of controlled trials with objectively measured outcomes (EarlyBird 54). BMJ 2012;**345**:e5888 doi: 10.1136/bmj.e5888[published Online First: Epub Date]|.

13. van Sluijs EM, McMinn AM, Griffin SJ. Effectiveness of interventions to promote physical activity in children and adolescents: systematic review of controlled trials. BMJ 2007;**335**(7622):703

14. Kipping R, Payne C, Lawlor DA. Randomised controlled trial adapting American school obesity prevention to England. Arch Dis Child 2008

15. McGoey T, Root Z, Bruner MW, Law B. Evaluation of physical activity interventions in children via the reach, efficacy/effectiveness, adoption, implementation, and maintenance (RE-AIM) framework: A systematic review of randomized and non-randomized trials. Prev Med 2016;**82**:8-19 doi: 10.1016/j.ypmed.2015.11.004[published Online First: Epub Date]|.

16. Arundell L, Fletcher E, Salmon J, Veitch J, Hinkley T. A systematic review of the prevalence of sedentary behavior during the after-school period among children aged 5-18 years. Int J Behav Nutr Phys Act 2016;**13**:93 doi: 10.1186/s12966-016-0419-1[published Online First: Epub Date]|.

17. Jago R, Rawlins E, Kipping RR, et al. Lessons learned from the AFLY5 RCT process evaluation: implications for the design of physical activity and nutrition interventions in schools. BMC Public Health 2015;**15**:946 doi: 10.1186/s12889-015-2293-1[published Online First: Epub Date]|.

For peer review only - http://bmjopen.bmj.com/site/about/guidelines.xhtml

18. Beets MW, Beighle A, Erwin HE, Huberty JL. After-school program impact on physical activity and fitness: a meta-analysis. Am J Prev Med 2009;**36**(6):527-37 doi: 10.1016/j.amepre.2009.01.033[published Online First: Epub Date]|.

19. Beets MW, Okely A, Weaver RG, et al. The theory of expanded, extended, and enhanced opportunities for youth physical activity promotion. Int J Behav Nutr Phys Act 2016;**13**(1):120 doi: 10.1186/s12966-016-0442-2[published Online First: Epub Date]|.

20. Beets MW, Weaver RG, Turner-McGrievy G, et al. Physical activity outcomes in afterschool programs: A group randomized controlled trial. Prev Med 2016;**90**:207-15 doi: 10.1016/j.ypmed.2016.07.002[published Online First: Epub Date]|.

21. Weaver RG, Beets MW, Huberty J, Freedman D, Turner-Mcgrievy G, Ward D. Physical activity opportunities in afterschool programs. Health promotion practice 2015;**16**(3):371-82 doi: 10.1177/1524839914567740[published Online First: Epub Date]|.

22. Jago R, Sebire SJ, Wood L, et al. Associations between objectively assessed child and parental physical activity: a cross-sectional study of families with 5-6 year old children. BMC Public Health 2014;**14**:655 doi: 10.1186/1471-2458-14-655[published Online First: Epub Date]|.

23. Jago R, Thompson JL, Sebire SJ, et al. Cross-sectional associations between the screen-time of parents and young children: differences by parent and child gender and day of the week. Int J Behav Nutr Phys Act 2014;**11**:54 doi: 10.1186/1479-5868-11-54[published Online First: Epub Date]|.

24. Cole TJ, Bellizzi MC, Flegal KM, Dietz WH. Establishing a standard definition for child overweight and obesity worldwide: international survey. BMJ 2000;**320**(7244):1240-3

25. Cole TJ, Freeman JV, Preece MA. Body mass index reference curves for the UK, 1990. Arch Dis Child 1995;**73**(1):25-9

For peer review only - http://bmjopen.bmj.com/site/about/guidelines.xhtml

For peer review only

26. Cain KL, Sallis JF, Conway TL, Van Dyck D, Calhoon L. Using accelerometers in youth physical activity studies: a review of methods. J Phys Act Health 2013;**10**(3):437-50

27. Basterfield L, Adamson AJ, Pearce MS, Reilly JJ. Stability of habitual physical activity and sedentary behavior monitoring by accelerometry in 6- to 8-year-olds. J Phys Act Health 2011;**8**(4):543-7

28. Jago R, Baranowski T, Baranowski J, et al. Fit for life Boy Scout badge: Outcome Evaluation of a troop & internet intervention. Prev Med 2006;**42**:181-87

29. Jago R, Baranowski T, Baranowski JC. Observed, GIS, and self-reported environmental features and adolescent physical activity. Am J Health Promot 2006;**20**(6):422-8

30. Evenson KR, Catellier DJ, Gill K, Ondrak KS, McMurray RG. Calibration of two objective measures of physical activity for children. Journal of sports sciences 2008;**26**(14):1557-65

31. Rubin DB. Multiple imputation after 18+ years. Journal of the American Statistical Association 1996;**91**(434):473-789

32. Mears R, Jago R. Effectiveness of after-school interventions at increasing moderate-to-vigorous physical activity levels in 5- to 18-year olds: a systematic review and meta-analysis. Br J Sports Med 2016 doi: 10.1136/bjsports-2015-094976[published Online First: Epub Date]|.

33. Brussoni M, Gibbons R, Gray C, et al. What is the Relationship between Risky Outdoor Play and Health in Children? A Systematic Review. Int J Environ Res Public Health 2015;**12**(6):6423-54 doi: 10.3390/ijerph120606423[published Online First: Epub Date]|.

34. Tremblay MS, Gray C, Babcock S, et al. Position Statement on Active Outdoor Play. Int J Environ Res Public Health 2015;**12**(6):6475-505 doi: 10.3390/ijerph120606475[published Online First: Epub Date]|.

For peer review only - http://bmjopen.bmj.com/site/about/guidelines.xhtml

35. Page A, Cooper A, Griew P, Davis L, Hillsdon M. Independent mobility in relation to weekday and weekend physical activity in children aged 10-11 years: The PEACH Project. Int J Behav Nutr Phys Act 2009;**6**:2

36. Page AS, Cooper AR, Griew P, Jago R. Independent mobility, perceptions of the built environment and children's participation in play, active travel and structured exercise and sport: the PEACH Project. Int J Behav Nutr Phys Act 2010;**7**:17

37. Cabinet Office. Childhood obesity: a plan for action. In: Prime Minister's Office DS, ed. London: Prime Minister's Office, 10 Downing Street, 2017.

38. Davies B, Wood L, Banfield K, Edwards MJ, Jago R. The provision of active after-school clubs for children in English primary schools: implications for increasing children's physical activity. Open Journal of Preventive Medicine 2014;**4**:598-605 doi: http://dx.doi.org/10.4236/ojpm.2014.47069[published Online First: Epub Date]|.

Figure 1 Predicted time spent in sedentary behaviour by type of activity using multiple imputation (N=1223)*

* Predictions were obtained from fully adjusted regression models (Model 2) including all Year 4 children (boys and girls) and are for a 9-year old boy with a BMI z-score of 0 and IMD score of 16. Predicted sedentary time for girls was approximately 15-17 mins/day higher (same additive effect across all categories of the exposure variable) depending on the regression model

Figure 2 Predicted time spent in moderate-to-vigorous physical activity by type of activity using multiple imputation (N=1223)*

* Predictions were obtained from fully adjusted regression models (Model 2) including all Year 4 children (boys and girls) and are for a 9-year old boy with a BMI z-score of 0 and IMD score of 16. Predicted time spent in moderate-to-vigorous physical activity for girls was approximately 13 mins/day lower (same additive effect across all categories of the exposure variable)

For peer review only

For peer review only

For peer review only - http://bmjopen.bmj.com/site/about/guidelines.xhtml

Organised physical activity, neighbourhood play and child physical activity

STROBE Statement—checklist of items that should be included in reports of observational studies

	Item No	Recommendation
**Title and abstract**	1	(*a*) Indicate the study's design with a commonly used term in the title or the abstract Title Page
		(*b*) Provide in the abstract an informative and balanced summary of what was done and what was found – Pages 2-3
**Introduction**		
Background/rationale	2	Explain the scientific background and rationale for the investigation being reported – Pages 5-6
Objectives	3	State specific objectives, including any prespecified hypotheses – Page 6.
**Methods**		
Study design	4	Present key elements of study design early in the paper Pages 6-7.
Setting	5	Describe the setting, locations, and relevant dates, including periods of recruitment, exposure, follow-up, and data collection Page 6.
Participants	6	(*a*) *Cohort study*—Give the eligibility criteria, and the sources and methods of selection of participants. Describe methods of follow-up<br>*Case-control study*—Give the eligibility criteria, and the sources and methods of case ascertainment and control selection. Give the rationale for the choice of cases and controls<br>*Cross-sectional study*—Give the eligibility criteria, and the sources and methods of selection of participants Page 7
		(*b*) *Cohort study*—For matched studies, give matching criteria and number of exposed and unexposed<br>*Case-control study*—For matched studies, give matching criteria and the number of controls per case
Variables	7	Clearly define all outcomes, exposures, predictors, potential confounders, and effect modifiers. Give diagnostic criteria, if applicable - Page 7-8
Data sources/ measurement	8*	For each variable of interest, give sources of data and details of methods of assessment (measurement). Describe comparability of assessment methods if there is more than one group Page 7-8
Bias	9	Describe any efforts to address potential sources of bias – Page 8
Study size	10	Explain how the study size was arrived at - Page 8
Quantitative variables	11	Explain how quantitative variables were handled in the analyses. If applicable, describe which groupings were chosen and why - Pages 8-9
Statistical methods	12	(*a*) Describe all statistical methods, including those used to control for confounding – Pages 8-9
		(*b*) Describe any methods used to examine subgroups and interactions – Pages 8-9
		(*c*) Explain how missing data were addressed - Pages 8-9
		(*d*) *Cohort study*—If applicable, explain how loss to follow-up was addressed<br>*Case-control study*—If applicable, explain how matching of cases and controls was addressed<br>*Cross-sectional study*—If applicable, describe analytical methods taking account of

For peer review only - http://bmjopen.bmj.com/site/about/guidelines.xhtml
For peer review only

		sampling strategy - Page 9
		(*e*) Describe any sensitivity analyses – Page 10

Continued on next page

For peer review only

**Results**		
Participants	13*	(a) Report numbers of individuals at each stage of study—eg numbers potentially eligible, examined for eligibility, confirmed eligible, included in the study, completing follow-up, and analysed Table 1
		(b) Give reasons for non-participation at each stage
		(c) Consider use of a flow diagram - NA
Descriptive data	14*	(a) Give characteristics of study participants (eg demographic, clinical, social) and information on exposures and potential confounders – Table 1
		(b) Indicate number of participants with missing data for each variable of interest - Table 1
		(c) *Cohort study*—Summarise follow-up time (eg, average and total amount)
Outcome data	15*	*Cohort study*—Report numbers of outcome events or summary measures over time
		Case-control study—Report numbers in each exposure category, or summary measures of exposure
		Cross-sectional study—Report numbers of outcome events or summary measures Table 1
Main results	16	(*a*) Give unadjusted estimates and, if applicable, confounder-adjusted estimates and their precision (eg, 95% confidence interval). Make clear which confounders were adjusted for and why they were included – Tables 2-4
		(*b*) Report category boundaries when continuous variables were categorized - Tables 2-4
		(*c*) If relevant, consider translating estimates of relative risk into absolute risk for a meaningful time period Discussion – Page 18
Other analyses	17	Report other analyses done—eg analyses of subgroups and interactions, and sensitivity analyses – Supplementary tables
**Discussion**		
Key results	18	Summarise key results with reference to study objectives – Page 21
Limitations	19	Discuss limitations of the study, taking into account sources of potential bias or imprecision. Discuss both direction and magnitude of any potential bias - Page 23
Interpretation	20	Give a cautious overall interpretation of results considering objectives, limitations, multiplicity of analyses, results from similar studies, and other relevant evidence – Page 22
Generalisability	21	Discuss the generalisability (external validity) of the study results Page 22
**Other information**		
Funding	22	Give the source of funding and the role of the funders for the present study and, if applicable, for the original study on which the present article is based – Page 24

*Give information separately for cases and controls in case-control studies and, if applicable, for exposed and unexposed groups in cohort and cross-sectional studies.

Note: An Explanation and Elaboration article discusses each checklist item and gives methodological background and published examples of transparent reporting. The STROBE checklist is best used in conjunction with this article (freely available on the Web sites of PLoS Medicine at http://www.plosmedicine.org/, Annals of Internal Medicine at http://www.annals.org/, and Epidemiology at http://www.epidem.com/). Information on the STROBE Initiative is available at www.strobe-statement.org.

For peer review only

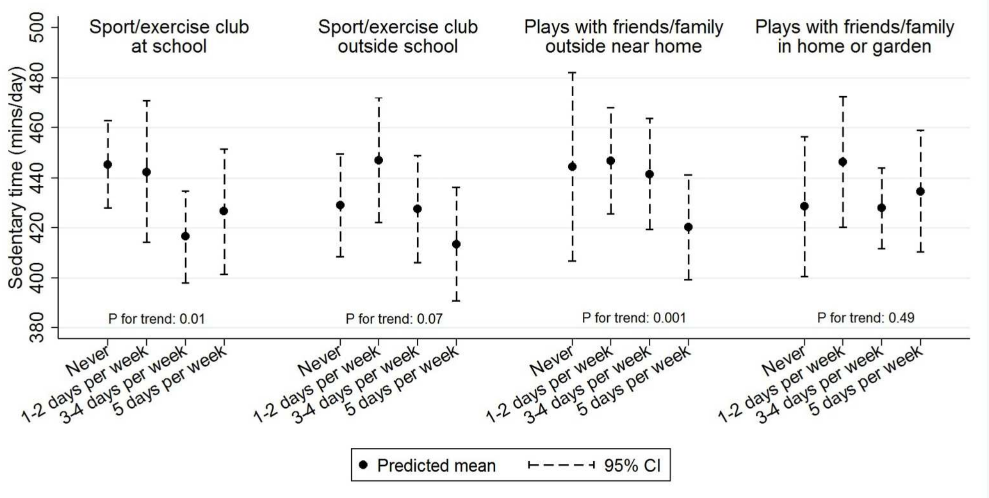

Figure 1 Predicted time spent in sedentary behaviour by type of activity using multiple imputation (N=1223)*

* Predictions were obtained from fully adjusted regression models (Model 2) including all Year 4 children (boys and girls) and are for a 9-year old boy with a BMI z-score of 0 and IMD score of 16. Predicted sedentary time for girls was approximately 15-17 mins/day higher (same additive effect across all categories of the exposure variable) depending on the regression model

159x79mm (300 x 300 DPI)

view only

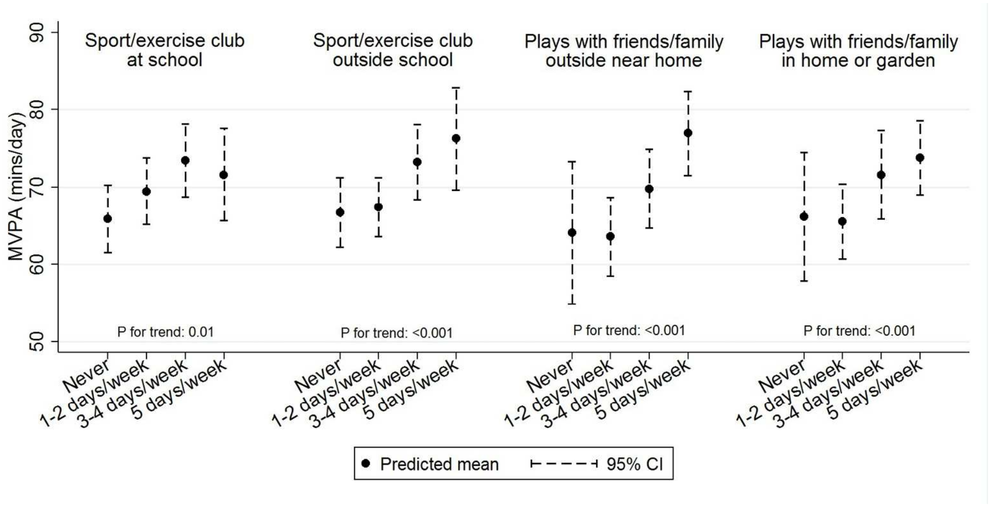

Figure 2 Predicted time spent in moderate-to-vigorous physical activity by type of activity using multiple imputation (N=1223)*

* Predictions were obtained from fully adjusted regression models (Model 2) including all Year 4 children (boys and girls) and are for a 9-year old boy with a BMI z-score of 0 and IMD score of 16. Predicted time spent in moderate-to-vigorous physical activity for girls was approximately 13 mins/day lower (same additive effect across all categories of the exposure variable)

159x79mm (300 x 300 DPI)

view only

Figure S1 Scatter plot of sedentary time by activity score in the observed data (N=1003)

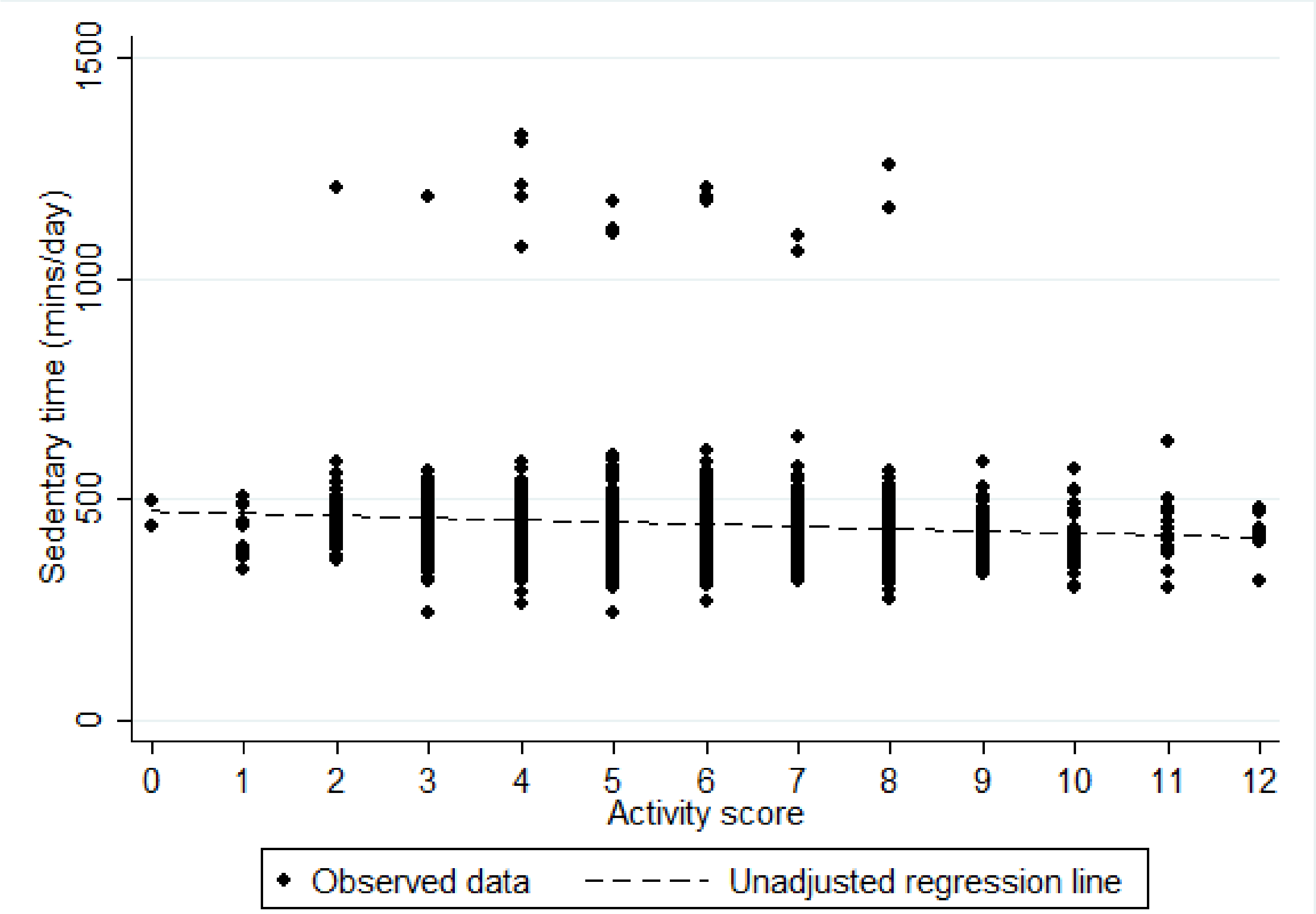

Figure S2 Scatter plot of time spent in moderate-to-vigorous physical activity by activity score in the observed data (N=1003)

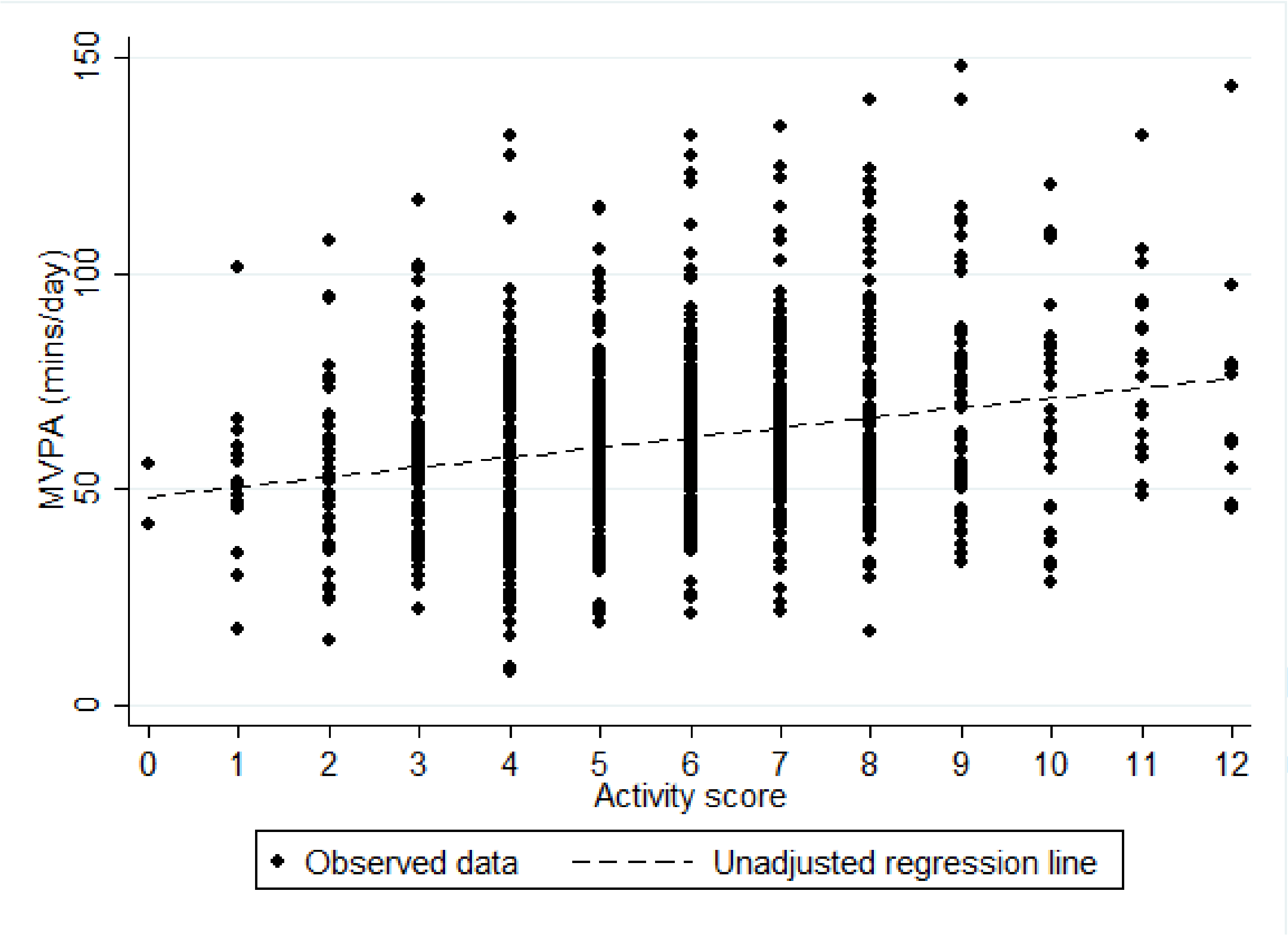

Table S1 Frequencies of sport/exercise club attendance and play outside and inside the home and mean activity score by gender in the observed data

		Boys<br>N (%)		Girls<br>N (%)		Chi-squared p-value for association
**Frequency child attends sport/exercise club at school**	Never<br>1-2 days/week<br>3-4 days/week<br>5 days/week	144 (25.9)<br>235 (42.3)<br>90 (16.2)<br>86 (15.5)		194 (29.4)<br>318 (48.2)<br>106 (16.1)<br>42 (6.4)		<0.001
**Frequency child attends sport/exercise club outside of school**	Never<br>1-2 days/week<br>3-4 days/week<br>5 days/week	85 (15.3)<br>289 (52.1)<br>121 (21.8)<br>60 (10.8)		165 (25.0)<br>321 (48.7)<br>132 (20.0)<br>41 (6.2)		<0.001
**Frequency child plays with friends/family outside near home**	Never<br>1-2 days/week<br>3-4 days/week<br>5 days/week	36 (6.6)<br>187 (34.1)<br>142 (25.9)<br>184 (33.5)		40 (6.1)<br>219 (33.4)<br>207 (31.6)<br>190 (29.0)		0.14
**Frequency child plays with friends/family in home/garden**	Never<br>1-2 days/week<br>3-4 days/week<br>5 days/week	53 (9.7)<br>184 (33.7)<br>142 (26.0)<br>167 (30.6)		62 (9.5)<br>230 (35.2)<br>179 (27.4)<br>182 (27.9)		0.76
		**Boys**		**Girls**		**T-test p-value for gender difference**
		N	Mean (SD)	N	Mean (SD)	
**Activity frequency score**		542	6.15 (2.42)	651	5.65 (2.16)	<0.001

For peer review only

Table S2 Child characteristics by frequencies of sport/exercise club attendance and play outside and inside the home and activity score in the observed data

		Age (years)		BMI z score		IMD score	
		Mean (SD)	N	Mean (SD)	N	Mean (SD)	N
**Frequency child attends sport/exercise club at school**	Never<br>1-2 days/week<br>3-4 days/week<br>5 days/week	9.03 (0.38)<br>9.05 (0.43)<br>9.00 (0.45)<br>8.99 (0.39)	1215	0.43 (1.14)<br>0.35 (1.05)<br>0.29 (1.02)<br>0.27 (1.13)	1202	17.30 (15.04)<br>15.54 (14.02)<br>14.15 (11.07)<br>16.66 (15.10)	1197
P-value for difference between categories*		0.31		0.36		0.07	
**Frequency child attends sport/exercise club outside of school**	Never<br>1-2 days/week<br>3-4 days/week<br>5 days/week	9.01 (0.40)<br>9.01 (0.43)<br>9.05 (0.41)<br>9.12 (0.39)	1214	0.47 (1.07)<br>0.37 (1.09)<br>0.23 (1.07)<br>0.30 (1.06)	1201	20.03 (16.06)<br>16.02 (14.12)<br>12.30 (10.25)<br>14.26 (13.64)	1196
P-value for difference between categories*		0.05		0.09		<0.001	
**Frequency child plays with friends/family outside near home**	Never<br>1-2 days/week<br>3-4 days/week<br>5 days/week	8.96 (0.47)<br>9.01 (0.42)<br>9.05 (0.41)<br>9.05 (0.40)	1205	0.48 (1.24)<br>0.31 (1.06)<br>0.39 (1.07)<br>0.33 (1.08)	1192	17.60 (16.02)<br>15.20 (13.83)<br>14.66 (12.48)<br>17.66 (15.08)	1187
P-value for difference between categories*		0.23		0.58		0.01	
**Frequency child plays with friends/family in home/garden**	Never<br>1-2 days/week<br>3-4 days/week<br>5 days/week	8.97 (0.38)<br>9.02 (0.45)<br>9.06 (0.41)<br>9.03 (0.39)	1199	0.50 (1.11)<br>0.29 (1.09)<br>0.40 (1.07)<br>0.32 (1.07)	1186	18.54 (15.94)<br>15.85 (13.95)<br>14.24 (12.79)<br>16.85 (14.55)	1181
P-value for difference between categories*		0.31		0.23		0.02	
		Correlation	P-value	Correlation	P-value	Correlation	P-value
**Activity frequency score**		0.04	0.13	-0.05	0.07	-0.07	0.02

For peer review only

Table S3 Pairwise comparisons of children's activity attendance frequencies in the multiple imputation data (N=1223)*

		**Frequency child attends sport/exercise club at school (%)**		**Frequency child attends sport/exercise club outside of school (%)**		**Frequency child plays with friends/family outside near home (%)**		**Frequency child plays with friends/family in home/garden (%)**	
		Up to 2 days/week	3 or more days/week	Up to 2 days/week	3 or more days/week	Up to 2 days/week	3 or more days/week	Up to 2 days/week	3 or more days/week
**Frequency child attends sport/exercise club at school**	Up to 2 days/week			76.3	23.7	42.7	57.3	47.3	52.7
	3 or more days/week			55.8	44.2	33.2	66.8	36.0	64.0
P for association				<0.001		0.003		0.001	
**Frequency child attends sport/exercise club outside of school**	Up to 2 days/week	79.0	21.0			40.2	59.8	46.8	53.2
	3 or more days/week	59.7	40.3			40.0	60.0	38.2	61.8
P for association		<0.001				0.94		0.006	
**Frequency child plays with friends/family outside near home**	Up to 2 days/week	78.0	22.0	71.0	29.0			65.1	34.9
	3 or more days/week	70.3	29.7	70.8	29.2			30.3	69.7
P for association		0.003		0.94				<0.001	
**Frequency child plays with friends/family in home/garden**	Up to 2 days/week	78.3	21.7	74.9	25.1	59.0	41.0		
	3 or more days/week	69.4	30.6	67.6	32.4	25.2	74.8		
P for association		0.001		0.006		<0.001			
**Total**		**73.4**	**26.6**	**70.8**	**29.2**	**40.2**	**59.8**	**44.3**	**55.7**

* Percentages presented are the proportions of children in each row that belong to each of the categories of child activity variables listed along the top of the table.

Table S4 Mean difference in the children's average sedentary minutes per day associated with different activities for those with complete data (N=987)*

Exposure		Sedentary time (minutes/day): mean difference (95% confidence interval)						
		All (N=987)		Boys (N=439)		Girls (N=548)		P for gender interaction
		Model 1	Model 2	Model 1	Model 2	Model 1	Model 2	
Frequency child attends sport/exercise club at school	Never (ref)	0	0	0	0	0	0	0.45
	1-2 days/week	-0.29 (-21.3, 20.7)	0.0 (-21.2, 21.3)	14.7 (-10.1, 39.5)	15.9 (-9.2, 41.0)	-11.4 (-39.7, 16.8)	-11.9 (-41.1, 17.3)	
	3-4 days/week	-26.3 (-40.8, -11.8)	-24.1 (-37.8, -10.4)	-13.8 (-31.5, 4.0)	-9.9 (-29.3, 9.4)	-36.0 (-59.5, -12.4)	-35.4 (-59.0, -11.7)	
	5 days/week	-16.0 (-35.3, 3.3)	-16.1 (-34.4, 2.3)	-7.1 (-29.7, 15.4)	-3.1 (-26.9, 20.6)	-22.0 (-54.6, 10.7)	-29.8 (-69.2, 9.66)	
P for trend		0.01	0.009	0.19	0.40	0.02	0.02	
Frequency child attends sport/exercise club outside of school	Never (ref)	0	0	0	0	0	0	0.73
	1-2 days/week	16.1 (-7.5, 39.7)	20.0 (-7.0, 47.0)	24.6 (-3.4, 52.7)	31.2 (0.6, 61.8)	10.8 (-19.3, 41.0)	12.9 (-20.0, 45.9)	
	3-4 days/week	-6.6 (-23.2, 10.1)	0.1 (-19.6, 19.8)	-3.2 (-28.8, 22.3)	5.6 (-17.7, 28.8)	-7.6 (-26.3, 11.1)	-2.0 (-26.7, 22.6)	
	5 days/week	-18.4 (-36.5, -0.2)	-13.8 (-33.8, 6.2)	-13.0 (-36.3, 10.4)	-5.9 (-28.8, 16.9)	-20.8 (-46.0, 4.3)	-18.2 (-46.4, 10.0)	
P for trend		0.009	0.05	0.02	0.03	0.08	0.28	
Frequency child plays with friends/family outside near home	Never (ref)	0	0	0	0	0	0	0.68
	1-2 days/week	4.5 (-26.7, 35.7)	6.0 (-27.5, 39.5)	16.3 (-13.4, 45.9)	20.2 (-10.1, 50.4)	-5.7 (-55.7, 44.3)	-7.1 (-59.0, 44.9)	
	3-4 days/week	-2.5 (-30.1, 25.0)	-0.6 (-31.6, 30.3)	8.3 (-23.1, 39.7)	13.0 (-19.4, 45.4)	-11.9 (-68.1, 44.3)	-13.2 (-71.3, 44.9)	
	5 days/week	-21.6 (-49.0, 5.7)	-21.9 (-50.4, 6.6)	-9.4 (-35.4, 16.6)	-6.4 (-32.8, 20.0)	-32.3 (-78.9, 14.2)	-36.4 (-86.0, 13.1)	
P for trend		<0.001	<0.001	0.08	0.08	0.009	0.01	
Frequency child plays with friends/family in home/garden	Never (ref)	0	0	0	0	0	0	0.86
	1-2 days/week	18.2 (-12.4, 48.8)	18.8 (-12.4, 50.0)	19.2 (-13.8, 52.2)	19.5 (-13.7, 52.8)	17.2 (-25.4, 59.9)	17.6 (-25.4, 60.6)	
	3-4 days/week	-4.5 (-27.3, 18.2)	-1.9 (-26.7, 22.9)	2.8 (-16.3, 21.9)	4.6 (-15.7, 25.0)	-10.1 (-47.3, 27.0)	-7.5 (-47.1, 32.1)	
	5 days/week	3.8 (-24.6, 32.2)	3.6 (-24.6, 31.8)	10.8 (-27.1, 48.8)	11.6 (-27.0, 50.2)	-2.2 (-43.0, 38.6)	-3.5 (-45.3, 38.3)	
P for trend		0.24	0.22	0.85	0.89	0.25	0.24	
Activity score (per unit)		-4.9 (-7.2, -2.7)	-4.7 (-6.9, -2.4)	-3.5 (-6.6, -0.4)	-2.9 (-6.0, 0.2)	-6.3 (-10.7, -2.0)	-6.6 (-11.2, -1.9)	0.28

* Model 1 is adjusted for age and gender; Model 2 is additionally adjusted for BMI and IMD score

For peer review only

Table S5 Mean difference in the children's average MVPA minutes per day associated with different activities for those with complete data (N=987)*

Exposure		Moderate-to-vigorous physical activity (minutes/day): mean difference (95% confidence interval)						
		All (N=987)		Boys (N=439)		Girls (N=548)		P for gender interaction
		Model 1	Model 2	Model 1	Model 2	Model 1	Model 2	
Frequency child attends sport/exercise club at school	Never (ref)	0	0	0	0	0	0	0.64
	1-2 days/week	3.2 (-0.4, 6.8)	3.1 (-0.5, 6.6)	3.3 (-3.3, 9.8)	2.7 (-3.7, 9.2)	3.1 (-0.5, 6.8)	3.1 (-0.6, 6.8)	
	3-4 days/week	6.7 (1.7, 11.8)	6.4 (1.4, 11.4)	8.0 (1.0, 15.1)	7.3 (0.1, 14.4)	5.7 (0.6, 10.7)	5.6 (0.5, 10.7)	
	5 days/week	5.3 (-1.3, 11.9)	5.1 (-1.5, 11.8)	6.6 (-1.4, 14.6)	5.8 (-2.2, 13.8)	3.2 (-5.0, 11.5)	3.1 (-5.5, 11.6)	
P for trend		0.02	0.03	0.02	0.05	0.08	0.10	
Frequency child attends sport/exercise club outside of school	Never (ref)	0	0	0	0	0	0	0.53
	1-2 days/week	1.8 (-1.9, 5.5)	1.5 (-2.4, 5.4)	4.3 (-3.2, 11.9)	3.6 (-4.4, 11.7)	0.4 (-3.5, 4.3)	0.4 (-3.5, 4.4)	
	3-4 days/week	7.3 (3.0, 11.5)	6.7 (2.4, 11.0)	10.5 (2.8, 18.2)	9.0 (1.4, 16.6)	5.3 (0.6, 10.0)	5.5 (0.9, 10.0)	
	5 days/week	10.5 (4.6, 16.5)	10.1 (3.9, 16.2)	14.3 (6.3, 22.3)	13.4 (4.8, 22.0)	7.3 (-0.8, 15.3)	7.3 (-0.6, 15.2)	
P for trend		<0.001	<0.001	<0.001	<0.001	0.01	0.008	
Frequency child plays with friends/family outside near home	Never (ref)	0	0	0	0	0	0	0.32
	1-2 days/week	0.7 (-4.1, 5.5)	0.4 (-4.3, 5.0)	1.2 (-7.4, 9.7)	0.5 (-8.1, 9.1)	0.4 (-6.0, 6.9)	0.3 (-6.3, 6.9)	
	3-4 days/week	5.2 (-0.8, 11.2)	4.9 (-1.0, 10.7)	6.7 (-3.5, 16.9)	6.0 (-4.0, 15.9)	4.0 (-3.6, 11.7)	3.9 (-3.8, 11.7)	
	5 days/week	9.9 (4.4, 15.4)	9.7 (4.2, 15.1)	13.8 (4.6, 23.0)	13.3 (4.0, 22.5)	6.5 (-0.5, 13.5)	6.4 (-0.7, 13.6)	
P for trend		<0.001	<0.001	<0.001	<0.001	0.004	0.005	
Frequency child plays with friends/family in home/garden	Never (ref)	0	0	0	0	0	0	0.40
	1-2 days/week	2.5 (-0.9, 6.0)	2.2 (-1.4, 5.8)	0.6 (-7.0, 8.2)	0.4 (-7.4, 8.2)	4.1 (-0.9, 9.2)	4.0 (-1.1, 9.1)	
	3-4 days/week	6.6 (2.4, 10.7)	6.3 (1.8, 10.8)	6.7 (-1.8, 15.3)	6.9 (-1.9, 15.7)	6.5 (0.4, 12.6)	6.4 (0.1, 12.7)	
	5 days/week	8.6 (4.6, 12.5)	8.4 (4.3, 12.6)	9.3 (1.6, 16.9)	9.1 (1.0, 17.2)	7.9 (2.5, 13.3)	7.8 (2.4, 13.3)	
P for trend		<0.001	<0.001	0.001	0.001	0.003	0.003	
Activity score (per unit)		2.13 (1.44, 2.82)	2.09 (1.37, 2.81)	2.58 (1.73, 3.43)	2.49 (1.55, 3.42)	1.67 (0.86, 2.47)	1.66 (0.85, 2.48)	0.07

* MVPA Moderate-to-vigorous physical activity; Model 1 is adjusted for age and gender; Model 2 is additionally adjusted for BMI and IMD score

For peer review only - http://bmjopen.bmj.com/site/about/guidelines.xhtml

For peer review only

Table S6 Odds ratios for achieving 60 minutes of MVPA per day associated with different activities for those with complete data (N=987)*

Exposure		Meeting government guideline: odds ratio (95% confidence interval)						
		All (N=987)		Boys (N=439)		Girls (N=548)		P for gender interaction
		Model 1	Model 2	Model 1	Model 2	Model 1	Model 2	
Frequency child attends sport/exercise club at school	Never (ref)	1	1	1	1	1	1	0.25
	1-2 days/week	1.27 (0.94, 1.71)	1.25 (0.93, 1.69)	0.97 (0.58, 1.63)	0.93 (0.55, 1.55)	1.57 (1.09, 2.25)	1.57 (1.09, 2.26)	
	3-4 days/week	1.77 (1.05, 2.97)	1.72 (1.03, 2.87)	1.97 (0.93, 4.18)	1.86 (0.88, 3.95)	1.65 (0.93, 2.91)	1.66 (0.94, 2.92)	
	5 days/week	1.63 (0.96, 2.77)	1.61 (0.95, 2.74)	1.49 (0.75, 2.96)	1.40 (0.70, 2.77)	1.73 (0.78, 3.85)	1.76 (0.78, 3.96)	
P for trend		0.03	0.03	0.06	0.10	0.07	0.07	
Frequency child attends sport/exercise club outside of school	Never (ref)	1	1	1	1	1	1	0.66
	1-2 days/week	1.32 (0.91, 1.91)	1.29 (0.88, 1.88)	1.53 (0.89, 2.62)	1.46 (0.83, 2.57)	1.19 (0.72, 1.97)	1.21 (0.74, 1.99)	
	3-4 days/week	2.06 (1.38, 3.07)	1.97 (1.32, 2.95)	2.56 (1.32, 4.98)	2.28 (1.18, 4.40)	1.77 (1.05, 2.98)	1.81 (1.09, 3.03)	
	5 days/week	2.89 (1.76, 4.73)	2.80 (1.69, 4.63)	4.13 (1.97, 8.68)	3.92 (1.82, 8.44)	2.09 (1.06, 4.12)	2.14 (1.10, 4.18)	
P for trend		<0.001	<0.001	<0.001	<0.001	0.006	0.004	
Frequency child plays with friends/family outside near home	Never (ref)	1	1	1	1	1	1	0.89
	1-2 days/week	1.16 (0.70, 1.92)	1.13 (0.70, 1.85)	1.03 (0.46, 2.29)	0.98 (0.43, 2.22)	1.40 (0.59, 3.30)	1.41 (0.60, 3.29)	
	3-4 days/week	1.94 (1.12, 3.35)	1.89 (1.12, 3.21)	1.59 (0.68, 3.75)	1.53 (0.65, 3.62)	2.43 (0.92, 6.45)	2.46 (0.94, 6.42)	
	5 days/week	2.14 (1.26, 3.65)	2.12 (1.26, 3.57)	2.01 (0.88, 4.63)	1.97 (0.85, 4.54)	2.45 (0.95, 6.36)	2.49 (0.97, 6.40)	
P for trend		<0.001	<0.001	0.007	0.005	0.007	0.006	
Frequency child plays with friends/family in home/garden	Never (ref)	1	1	1	1	1	1	0.30
	1-2 days/week	1.33 (0.88, 2.00)	1.30 (0.86, 1.97)	0.96 (0.51, 1.78)	0.94 (0.50, 1.77)	1.92 (0.90, 4.11)	1.94 (0.91, 4.12)	
	3-4 days/week	1.67 (1.10, 2.54)	1.64 (1.07, 2.52)	1.33 (0.66, 2.69)	1.36 (0.66, 2.82)	2.15 (0.96, 4.79)	2.16 (0.97, 4.80)	
	5 days/week	1.86 (1.21, 2.86)	1.85 (1.20, 2.86)	1.68 (0.86, 3.25)	1.67 (0.84, 3.32)	2.18 (1.04, 4.60)	2.20 (1.04, 4.62)	
P for trend		0.002	0.002	0.008	0.008	0.06	0.06	
Activity score (per unit)		1.19 (1.12, 1.26)	1.19 (1.12, 1.26)	1.20 (1.11, 1.30)	1.19 (1.10, 1.30)	1.18 (1.09, 1.28)	1.18 (1.09, 1.28)	0.79

* MVPA Moderate-to-vigorous physical activity; Model 1 is adjusted for age and gender; Model 2 is additionally adjusted for BMI and IMD score

For peer review only
